# Optically driven intelligent computing with ZnO memristor

**DOI:** 10.1016/j.fmre.2022.06.019

**Published:** 2022-07-25

**Authors:** Jing Yang, Lingxiang Hu, Liufeng Shen, Jingrui Wang, Peihong Cheng, Huanming Lu, Fei Zhuge, Zhizhen Ye

**Affiliations:** a Ningbo Institute of Materials Technology and Engineering, Chinese Academy of Sciences, Ningbo 315201, China; b Center of Materials Science and Optoelectronics Engineering, University of Chinese Academy of Sciences, Beijing 100029, China; c Center for Excellence in Brain Science and Intelligence Technology, Chinese Academy of Sciences, Shanghai 200072, China; d Institute of Wenzhou, Zhejiang University, Wenzhou 325006, China; e State Key Laboratory of Silicon Materials, School of Materials Science and Engineering, Zhejiang University, Hangzhou 310027, China

**Keywords:** Memristor, All-optically controlling, ZnO thin film, Artificial vision, Nonvolatile neuromorphic computing, Logic-in-memory

## Abstract

Artificial vision is crucial for most artificial intelligence applications. Conventional artificial visual systems have been facing challenges in terms of real-time information processing due to the physical separation of sensors, memories, and processors, which results in the production of a large amount of redundant data as well as the data conversion and transfer between these three components consuming most of the time and energy. Emergent optoelectronic memristors with the ability to realize integrated sensing-computing-memory (ISCM) are key candidates for solving such challenges and therefore attract increasing attention. At present, the memristive ISCM devices can only perform primary-level computing with external light signals due to the fact that only monotonic increase of memconductance upon light irradiation is achieved in most of these devices. Here, we propose an all-optically controlled memristive ISCM device based on a simple structure of Au/ZnO/Pt with the ZnO thin film sputtered at pure Ar atmosphere. This device can perform advanced computing tasks such as nonvolatile neuromorphic computing and complete Boolean logic functions only by light irradiation, owing to its ability to reversibly tune the memconductance with light. Moreover, the device shows excellent operation stability ascribed to a purely electronic memconductance tuning mechanism. Hence, this study is an important step towards the next generation of artificial visual systems.

## Graphical abstract

**Figure:**
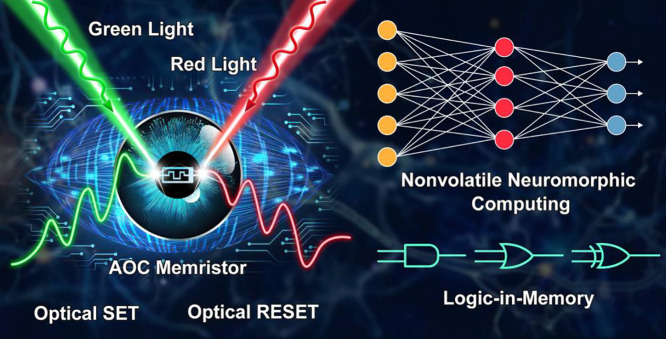

## 1 Introduction

A conventional artificial vision system is composed of the visual sensor, the information processing unit and the memories, which are physically separated from each other. The analogue information captured by the visual sensor is first converted to digital signals, and then temporarily stored in the memory, before being transferred to the processing unit for pattern recognition. As a result, a large amount of redundant data will be produced, which greatly limits the processing efficiency as well as occupying massive storage resources [1]. Furthermore, the data conversion and transfer between the sensor, memory and processor will consume most of the time and energy partly due to a limited bandwidth [2]. Some techniques have been employed to improve these shortcomings such as increasing the transmission bandwidth [3] and preprocessing the visual data to alleviate redundant data [[4], [5]] (*e.g*. background subtraction). However, these improvements in the performance of conventional artificial vision systems are increasingly unable to meet the demands for real-time processing of explosively growing visual information. A reasonable solution is to use multifunctional optoelectronic devices that can realize integrated sensing-computing-memory (ISCM) given the elimination of data conversion and transfer processes between sensors, memories, and processors as well as significantly reduced redundant data [2]. An artificial vision system composed of ISCM devices is expected to process the visual information in real-time.

Optoelectronic memory devices can generally perform calculations *in situ, i.e*., computations can be carried out in the memory, as well as being sensitive to light, thus endowing them with ISCM functions [[6], [7], [8], [9], [10]]. For an ideal ISCM device, the computing is driven directly by external optical signals since it is the most beneficial mode for increasing calculation speed and decreasing power consumption [[11], [12], [13]]. Till now, ISCM devices have been realized based on different structures such as memristors [[2], [6], [7], [14], [15], [16], [17], [18], [19], [20], [21]] and transistors [[8], [9], [22], [23], [24], [25], [26], [27]]]. Memristors have a simple two-terminal structure that is favourable for high-density integration, in addition to high operation speed [[28], [29]]. Recently, optoelectronic memristors possessing the capability of ISCM have been widely reported [[2], [6], [7], [14], [15], [16], [17], [18], [19], [20], [21]]. However, due to monotonic change (*i.e*., increase) of memconductance upon light irradiation in most of those devices, only primary-level computing could be performed with external light signals, such as simulations of basic synaptic functions and simple pre-processing of visual information. To conduct advanced computation including nonvolatile neuromorphic computing and complete Boolean logic functions, it was necessary to introduce additional electrical signals which were used to decrease the memconductance [[2], [14], [15], [16], [17], [18], [19]]. A combination of external optical and electrical signals is unfavourable to real-time processing of visual information with high energy efficiency.

In this work, we propose a high-level ISCM device based on an all-optically controlled (AOC) memristor. The device has a simple structure of Au/ZnO/Pt. Unlike previously reported memristive ISCM devices, it can perform advanced computing tasks only by light irradiation thanks to its ability to reversibly tune the memconductance with light. This device shows excellent operation stability due to a purely electronic tuning mechanism of memconductance. Therefore, our AOC memristive ISCM device has great application potential for designing next generation artificial visual systems, which have the ability to detect, process, and store visual information in real-time.

## 2 Materials and experimental methods

### 2.1 Material growth

Polycrystalline ZnO thin films were deposited on Pt/Ti/SiO_2_/Si, ITO coated SiO_2_/Si, and quartz substrates at room temperature by RF magnetron sputtering of a ZnO ceramic target of 99.99% purity in pure Ar atmosphere. The sputtering power was 60 W and the pressure was 0.5 Pa. The structure of the as-deposited ZnO films was checked by an X-ray diffractometer (XRD, D8 Advance). The thickness was determined to be about 50 nm via variable angle spectroscopic ellipsometry (M-2000 DI, J. A. Woollam Co., Inc.). The resistivity was measured with a Hall effect measurement system (Lake Shore 8400) using the van der Pauw method. The as-deposited ZnO films showed an electron concentration of 10^14^ cm^–3^ and a resistivity of 10^4^ Ω cm. Transmittance spectra were measured by a UV–visible–IR spectrophotometer (Lambda 950, PerkinElmer). Photoluminescence spectra were measured via a confocal microscopic Raman spectrometer (Renishaw inVia Reflex, 325 nm). These measurements were performed at room temperature in the air.

### 2.2 Device fabrication and characterization

Au top-electrodes with a thickness of 10 nm and a diameter of 100 *μ*m were deposited onto ZnO films at room temperature by electron beam evaporation with *in situ* metal shadow masks. Ti and Cu top-electrodes with a thickness of 3 nm and 20 nm were deposited by the same method. To avoid oxidization of Ti and Cu, a 10 nm thick Au protection layer was deposited onto the Ti and Cu electrodes. ITO top-electrodes with a thickness of 100 nm and a diameter of 100 *μ*m were deposited at room temperature by RF magnetron sputtering of an ITO (In_2_O_3_:SnO_2_ = 5:1, molar ratio) ceramic target of 99.99% purity in pure Ar. The sputtering power was 60 W and the pressure was 0.5 Pa. Electrical and optoelectronic measurements were conducted at room temperature in air by a Keithley 4200 semiconductor parameter analyzer equipped with a monochromatic light source (Omni-λ 3007). Voltage was applied to the top electrode (Au or ITO) with the bottom electrode (Pt or ITO) grounded. Light was injected into the device through the top electrode. Unless otherwise specified, the light power density was maintained at 36 *μ*W/cm^2^. The fabricated device was in a high memconductance state (HMS) most likely due to inevitable exposure to light from the environment and the microscope since the device is sensitive to visible light. Such a HMS could be restored to the initial low memconductance state (LMS) via bias voltage or light irradiation. Unless otherwise specified, the electrical and optoelectronic measurements were based on initialized devices.

## 3 Results and discussion

### 3.1 Memristive switching behavior

**Fig. 1:**
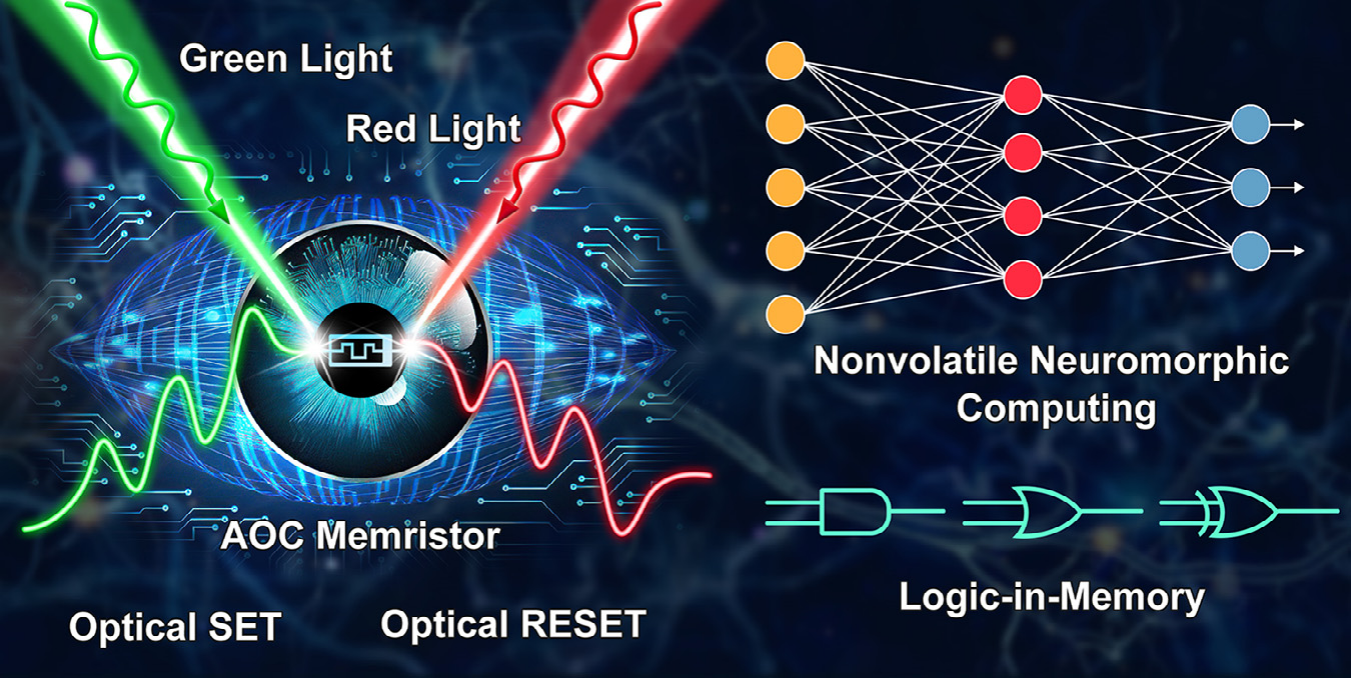
**Schematic of a high-level ISCM device based on AOC memristor.** The continuous increase/decrease of memconductance is realized upon green/red light irradiation (*i.e.*, optical SET/RESET). This ISCM device can perform nonvolatile neuromorphic computing and Boolean logic operations, which are driven directly by external optical signals

An AOC memristive ISCM device is schematically illustrated in Fig. 1. We used polycrystalline ZnO with a wide band gap of 3.2 eV as the active layer (Fig. S1). ZnO fabricated by various methods has been widely used as the active layer of memristive devices [[15], [30]]. Herein, the ZnO thin film was sputtered in pure Ar gas. The device demonstrated a nonpolar memristive switching behavior, *i.e.*, from an HMS to an LMS when measured in the dark (Fig. S2a). To investigate the switching mechanism, the metal electrodes were replaced by a transparent conducting oxide (Sn-doped In_2_O_3_ (ITO)), which formed quasi-Ohmic contact with ZnO given that the electron affinity of ZnO (4.2 eV) and the work function of ITO (4.3 eV) have similar values [[31], [32]]. No memristive switching phenomenon was observed in ITO/ZnO/ITO (Fig. S2b). This meant that the switching occurred at the Au/ZnO or the ZnO/Pt interface. We also found that the memconductance strongly depended on the device size (*i.e.*, the diameter of the Au electrode) for both HMSs and LMSs (Fig. S2c). It demonstrates that a homogeneous memristive switching occurred at the metal/ZnO interface [[6], [30]].

It is known that ZnO is naturally an n-type semiconductor because of the existence of intrinsic donor defects, such as oxygen vacancies (*V*_O_s) and zinc interstitials (Zn*_i_*s) [[30], [32]]. Herein, for the sake of simplicity, we consider only *V*_O_s. When ignoring the interface states, two back-to-back Schottky junctions at Au/ZnO and ZnO/Pt interfaces would be formed given that the electron affinity of ZnO was lower than the work functions of Au (5.1 eV) and Pt (5.65 eV) (Fig. S3a). It is worth mentioning that the ideal Schottky barrier widths of Au/ZnO and Pt/ZnO were estimated to be 1.96 μm and 2.69 μm, respectively, which were much larger than 50 nm of the ZnO film thickness. Hence, the Schottky junctions should occupy the whole thickness of ZnO.

In an equilibrium state without bias voltage, the numbers of electrons tunneling through the junction along both directions were equal and the net current was zero (Fig. S3b). When applying a bias voltage, there was a flow of electrons into ZnO (Fig. S3c). Part of the electrons were trapped by ionized oxygen vacancies (mainly *V*_O_^2+^s), which then transformed into neutral *V*_O_s*.* A decrease in the number of *V*_O_^2+^s resulted in an increase in the difference between the conduction band minimum (*E*_C_) and the Fermi energy (*E*_F_) according to *E*_C_ – *E*_F_ = *kT* ln(*N*_C_/*N*_D_), where *k* is the Boltzman constant, *T* the absolute temperature, *N*_C_ the effective density of states in the conduction band, and *N*_D_ the donor (*i.e., V*_O_^2+^s) density. As shown in Fig. S3c, the increase in *E*_C_ – *E*_F_ results in a decreased curvature of the energy band, which decreases the probability of electron tunneling and thus lowers the memconductance. It deserves mentioning that the voltage bias induced electrons from the Pt (or Au) electrode into ZnO, and at the same time, the same number of electrons flowed out of ZnO via the Au (or Pt) electrode. This implies that there were no additional electrons in the ZnO layer or in the Au/ZnO/Pt device. The electrons captured by *V*_O_^2+^s could be considered to be the free electrons already present in ZnO. Therefore, it follows that the density of free electrons in ZnO decreased, which also contributed to the lowered memconductance.

### 3.2 Optical SET and RESET behaviors

**Fig. 2:**
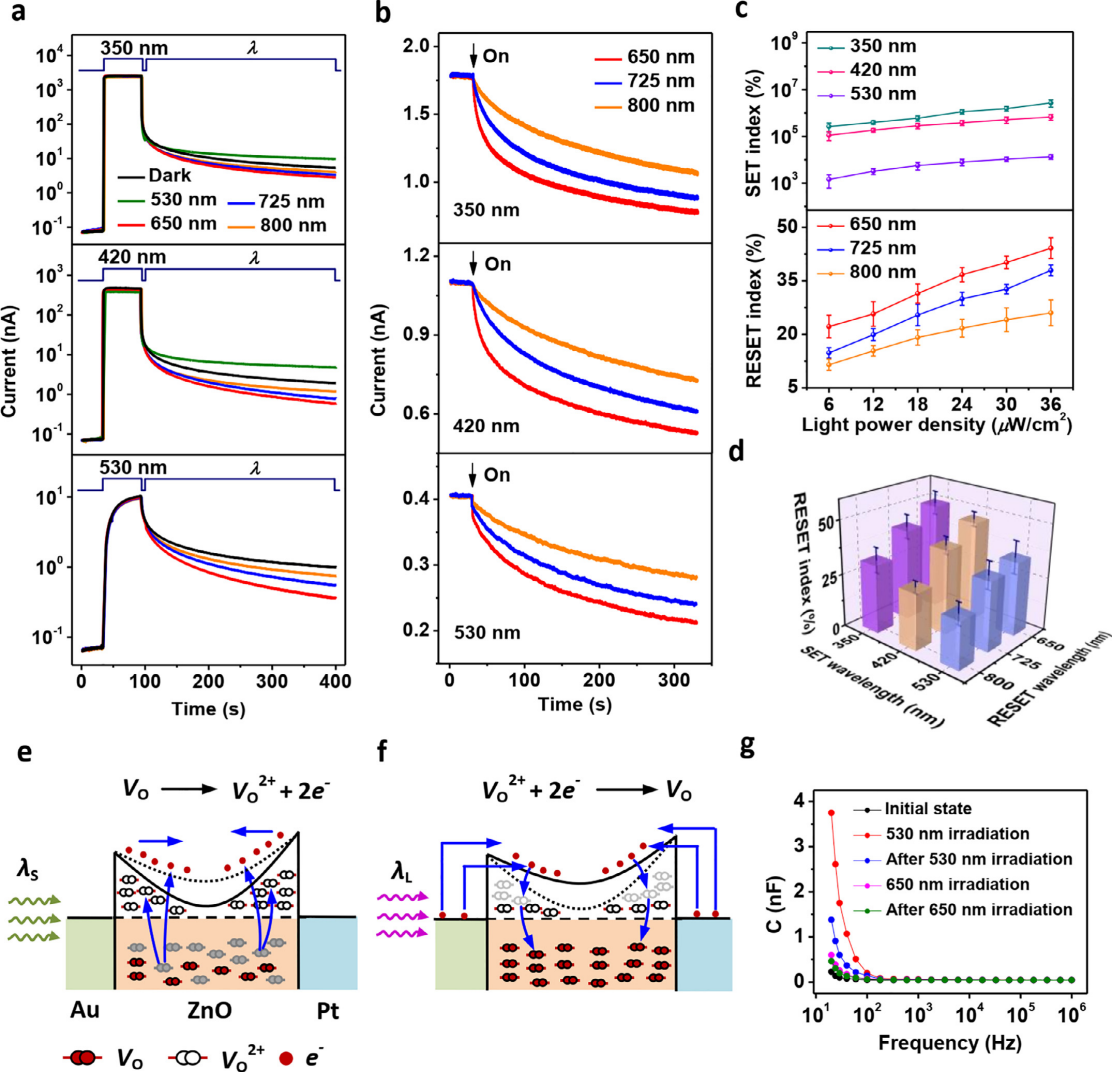
**Optical SET and RESET behaviors**. (a) Dependence of the photocurrent response on the wavelength (*λ*) of the irradiated light. The device was first irradiated with 350, 420, and 530 nm light (duration (*D*) = 60 s). (b) Optical RESET behavior upon exposure to light of various wavelengths (*D* = 300 s). The device was first set to HMSs by irradiating it with 350, 420, and 530 nm light (*D* = 60 s). (c) Dependence of the SET (top panel) and RESET (bottom panel) indexes on light power density. For the RESET operation, the device was first set to an HMS with 530 nm light, followed by irradiating it with light of long wavelengths 30 minutes after the initial short-wavelength light illumination. (d) Dependence of the RESET index on wavelength of the light used for the initial SET and the subsequent RESET operations. (e) Equilibrium energy band diagram of the Au/ZnO/Pt device after irradiation with short-wavelength light (*λ*_S_). The *V*_O_ ionization reaction is also schematically illustrated (blue arrows). The black dotted line indicates the positions of *E*_C_ before irradiation. (f) Equilibrium energy band diagram after irradiation with long wavelength light (*λ*_L_). The electron tunneling and jumping processes as well as the subsequent *V*_O_^2+^ neutralization reaction are also schematically illustrated (blue arrows). The black dotted line indicates the positions of *E*_C_ before irradiation. (g) Capacitance‒frequency characteristics of the AOC memristor. The device was first at the initial LMS (black curve), and then exposed to 530 nm light (red curve), and afterwards kept in the dark for 30 minutes (blue curve); subsequently, the device was exposed to 650 nm light (pink curve), and finally kept in the dark for 30 minutes (green curve). In (a-d, g), the light power densities were maintained at 36 *μ*W/cm^2^ and the current values were measured at 10 mV. Note that both the *V*_O_ ionization and *V*_O_^2+^ neutralization reactions and electron motion actually occurred under nonequilibrium conditions.

The Au/ZnO structure has a mean transmittance of > 60% for light wavelengths from 350 nm to 1000 nm (Fig. S4). This suggests that its memconductance could be modulated via light irradiation. Light was injected into the device through the top electrode (Au). Upon illumination with short-wavelength lights (350 nm and 420 nm), the device current instantaneously increased and reached to a saturation value (Fig. 2a, top and middle panels). After illumination, the device showed a persistent photocurrent (PPC) phenomenon (see the black curve). Green light (530 nm) irradiation caused a gradual increase in the current followed by a PPC (Fig. 2a, bottom panel, black curve). PPC is known to be an intrinsic phenomenon in most optoelectronic devices, which can be understood as follows: irradiation with light of appropriate wavelengths results in an increase in the current; after irradiation, although a current decay occurs, the device cannot be restored to its initial conductance state before irradiation, *i.e.*, the conductance state after irradiation is nonvolatile. Therefore, relatively short-wavelength light (350 nm, 420 nm, and 530 nm) could switch the device from LMS to HMS, referred to as the SET operation. On the other hand, when the device was irradiated with long-wavelength light (650 nm, 725 nm, and 800 nm), very weak or even no current change was observed (Fig. S5).

After irradiating the device with 350 nm and 420 nm light, subsequent irradiation with 530 nm light resulted in an increase in the photocurrent, whereas 650 nm, 725 nm and 800 nm light gave rise to a decrease in the current, compared to the case without subsequent irradiation (Fig. 2a, top and middle panels). Similarly, for the device irradiated with 530 nm light, subsequent irradiation with relatively long-wavelength light induced a decrease in the current (Fig. 2a, bottom panel). These observations indicate that long-wavelength light could be used to switch the device from HMS to LMS, which is called the RESET operation. To demonstrate the RESET behavior more clearly, the device was first set to HMSs with 350 nm, 420 nm, and 530 nm light, followed by irradiating it with light of long wavelengths 30 minutes after the initial short-wavelength light illumination (Fig. 2b). The purpose of the 30-minute wait was to ensure that the HMSs were relatively stable. A clear decrease in current could be observed upon the 650 nm, 725 nm and 800 nm light exposures.

SET and RESET indexes were used to quantitatively study the optical SET and RESET efficiencies. The index values were calculated by the formula (|*I*_1_ – *I*_2_|/*I*_1_) × 100%, where *I*_1_ and *I*_2_ are the device currents before and after irradiation, respectively. As demonstrated in Fig. 2c, both the SET and RESET indexes increased with increasing light power density. Fig. 2d illustrates the dependence of RESET indexes on the light wavelength for the initial SET operation and the following RESET operation. Evidently, the RESET index increased with a decrease in wavelength of the light for both SET and RESET.

As mentioned previously, the curvature of the conduction band dominated the device memconductance. It could be deduced that this curvature played a key role in the optical SET operation (Fig. 2e). Specifically, light induced an ionization reaction in which neutral *V*_O_ transformed into *V*_O_^2+^s. An increase in the number of *V*_O_^2+^s caused a decrease in *E*_C_ – *E*_F_ and then led to an increased curvature of the conduction band, thus heightening the memconductance. The nonvolatility of the light-induced memconductance states is likely due to the following reasons: i) the free electrons generated in the Schottky barrier region were pulled away by the built-in electric field and therefore could not recombine with *V*_O_^2+^s, and ii) an energy barrier originating from the outward relaxation of bonds around the oxygen vacancy sites must be overcome to neutralize *V*_O_^2+^s [33]. The visible light (*e.g.*, 530 nm) response of such wide band gap ZnO is due to an abundance of *V*_O_s with a wide distribution of energy levels given that ZnO was deposited in pure Ar [[34], [35]]. It is also supported by photoluminescence measurement (Fig. S6), in which broad deep emission bands ranging from 480 to 900 nm were observed. In addition, it has been reported that *V*_O_s in ZnO could have energy levels ranging from 0.2 eV to 1.3 eV below the conduction band minimum [36]. By contrast, we found that the Au/ZnO/Pt device with ZnO deposited at an Ar/O_2_ mixture atmosphere showed volatile photoconductance behaviors instead of nonvolatile PPC (Fig. S7).

Contrary to the optical SET, an optical RESET is expected to result from a decreased curvature of the conduction band due to a reduced density of *V*_O_^2+^. As mentioned previously, no significant photocurrent was generated by irradiation with 650 nm, 725 nm, and 800 nm light for the device in LMS (Fig. S5). Therefore, we can deduce that for the device after the optical SET, *i.e.*, in an HMS, ionization of *V*_O_s could be ignored under irradiation with such long-wavelength light given a lower density of *V*_O_s in ZnO compared to the case without the optical SET (in LMS). It has been reported that in a metal/oxide/metal junction [[37], [38]], electrons in metal could be injected into the oxide via internal photoemission or photoassisted tunnelling during light irradiation. Hence, a possible explanation for the optical RESET is that electrons in the metal electrode entered the conduction band of ZnO upon long-wavelength light irradiation, as schematically illustrated in Fig. 2f. Part of these electrons neutralized *V*_O_^2+^s. Thus, a decreased density of *V*_O_^2+^s resulted in a decreased curvature of the conduction band. This explanation is well supported by the strong dependence of the RESET efficiency on the light power density (shown in Fig. 2c, bottom panel) and on the RESET wavelength (shown in Fig. 2d). The reason can be understood as follows: irradiation with a relatively short RESET wavelength having relatively high photon energy or with a relatively high light power density generated more electrons injected from metal to ZnO; it follows that more electrons neutralized *V*_O_^2+^s, resulting in a lower density of *V*_O_^2+^s.

We also measured the device capacitance at different irradiation conditions (Fig. 2g). Capacitance of the device initially increased when irradiated with 530 nm light, but decreased upon a second irradiation with 650 nm light. The depletion layer capacitance *C*_j_ could be calculated as *C*_j_ = *ε*_0_*ε*_r_/*L*, where *ε*_0_ is the vacuum dielectric constant, *ε*_r_ the relative dielectric constant of the depletion layer, and *L* the depletion layer width (50 nm). It has been reported that a charged dielectric material showed a relatively high *ε*_r_ [39]. Hence, the dependence of the device capacitance on light irradiation confirms that the 530 nm irradiation resulted in an increase in the *V*_O_^2+^ density, whereas the 650 nm irradiation led to a decrease in the *V*_O_^2+^ density.

We believe that during the optical SET process, short-wavelength light with relatively high photon energy also induced internal photoemission or photoassisted tunnelling of electrons from the metal electrode, thus causing neutralization of *V*_O_^2+^s. The reason why ionization of *V*_O_s dominated during the SET process may be as follows: *V*_O_ ionization was apt to occur under short-wavelength irradiation whereas neutralization of *V*_O_^2+^s was limited by the number of electrons injected from metal to the conduction band of ZnO via internal photoemission or photoassisted tunnelling; given that the number of injected electrons was severely limited by the Schottky barrier (Fig. 2f), *V*_O_^2+^ neutralization should play a non-dominating role in the optoelectronic response. It deserves mentioning that the device was driven into a nonequilibrium state where the quasi-Fermi level of electrons of ZnO moved toward the conduction band upon irradiation with short-wavelength light since additional free electrons were generated in ZnO. Due to the difference of Fermi levels between ZnO and metal, the electrons would flow from ZnO to metal spontaneously. These electrons would, to some extent, compensated the electrons injected from metal to ZnO via internal photoemission or photoassisted tunneling, thus leading to further weakened neutralization reaction of *V*_O_^2+^s.

### 3.3 AOC memristor

**Fig. 3:**
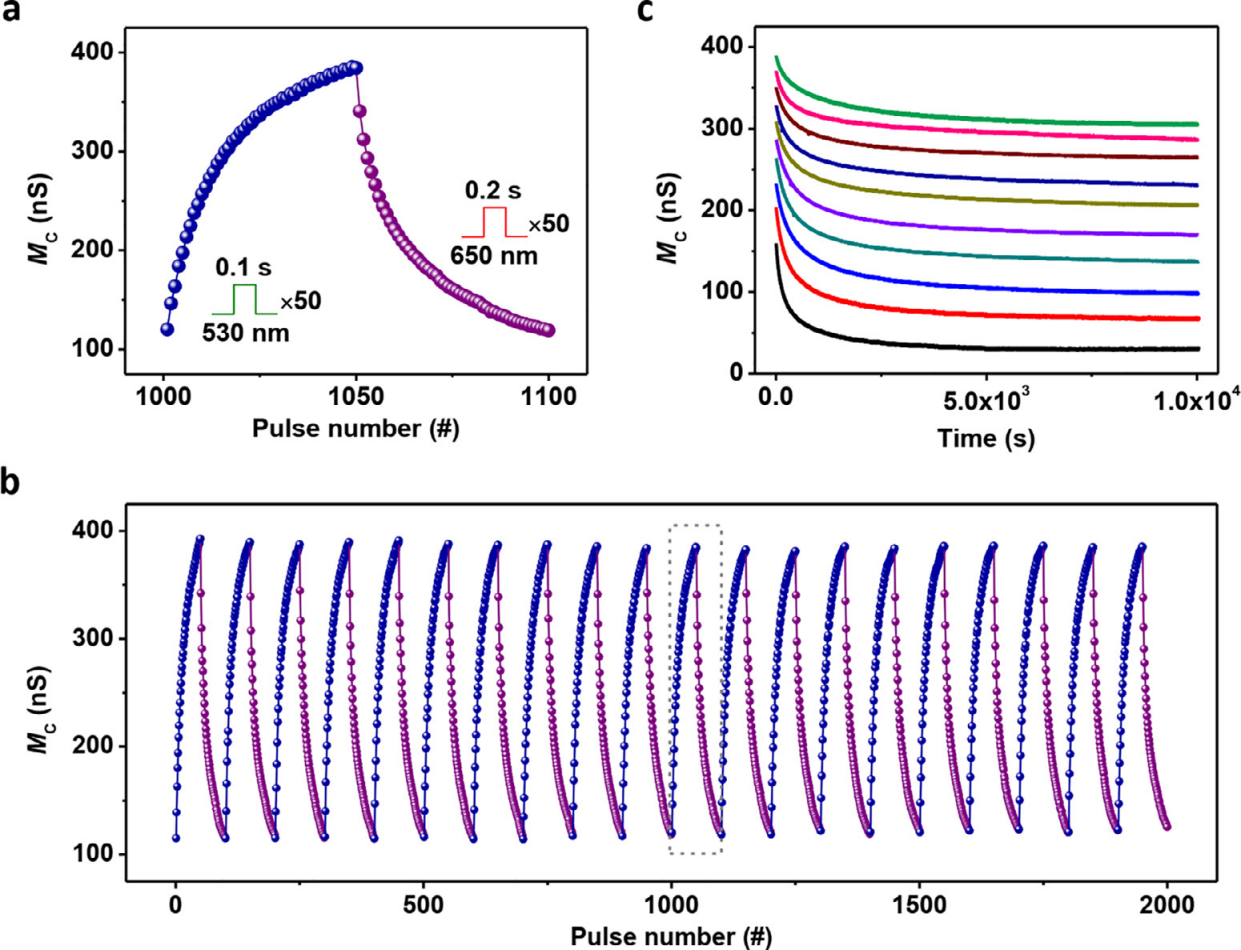
**All-optically tuning of memconductance (*****M***_**C**_**).** (a) Reversible modulation of memconductance by using 50 green light pulses (*D* = 100 ms and interval (*I*) = 1 s) and 50 red light pulses (*D* = 200 ms and *I* = 1 s). (b) 20 successive memconductance increase/decrease cycles. An enlarged view of the eleventh cycle (gray rectangle) is shown in (a). (c) Retention performance of 10 memconductance states induced by light. The device was first set to a low memconductance state followed by ten SET operations with different numbers of 530 nm light pulses. After each SET operation and following retention test, the device was set to the initial low memconductance state again. In (a) and (b), the memconductance values during the SET and RESET processes were measured just before applying the next pulse, *i.e.*, the delay time between applying light pulse and measuring the memconductance was equal to the interval time of light pulses. All the memconductance values were measured at 10 mV.

Based on the optical SET and RESET operations, we can realize the reversible tuning of memconductance by applying only optical excitation. Fig. 3a exhibits a continuous increase (SET) and decrease (RESET) in the memconductance upon successive light pulses of 530 and 650 nm, respectively. The ON/OFF ratio was calculated to be about 3.2. Herein, ON/OFF ratio was calculated as the ratio of highest memconductance to lowest memconductance. Fig. 3b shows the results of 20 successive memconductance increase/decrease cycles. The retention behaviors of ten states are illustrated in Fig. 3c. The memconductance initially showed a slow decay and then remained almost stable. All the states could be clearly distinguished even after 10^4^ s, indicating nonvolatility of the light-induced memconductance states. The decay curves were fitted using exponential functions to further verify their nonvolatility (Fig. S8). The fitting results demonstrated that the memconductance could be maintained above a certain value with time, thus confirming the nonvolatile memconductance states. It deserves mentioning that the observed conductance decay is very similar to the PPC effect, an intrinsic phenomenon observed in most optoelectronic devices. Such decay could be explained as follows: the irradiated device was in a nonequilibrium state; electrons in the metal electrode tended to tunnel through the Schottky barrier and were captured by *V*_O_^2+^s, thus resulting in barrier widening. To study the device-to-device variation of the light-induced memconductance states, memconductance increase/decrease cycles from 20 randomly selected devices were measured (Fig. S9). The lowest and highest memconductance states showed a memconductance range of 95–126 nS and 310–390 nS, respectively. The ON/OFF ratios were in a range of 2.7–4.1.

To compare the memconductance tuning performance between optically and electrically controlled memristors, we prepared Ti/ZnO/Pt and Cu/ZnO/Pt devices in which the ZnO layer was deposited at the same parameters as the AOC memristor (Au/ZnO/Pt). It has been reported that Ti/ZnO/Pt and Cu/ZnO/Pt demonstrated memristive switching due to electrically controlled electron trapping/detrapping and Cu nanofilament rupture/rejuvenation, respectively [[30], [40]]. After an electroforming process, the Au/ZnO/Pt showed memristive switching based on electrically controlled rupture and rejuvenation of conducting nanofilaments composed of oxygen vacancies [41]. Fig. S10–S12 illustrate electric-induced memristive behaviors of the Ti/ZnO/Pt, Cu/ZnO/Pt and electroformed Au/ZnO/Pt devices, respectively. By comparing the single memconductance increase/decrease cycles in Fig. 3 and S10–S12, we could observe that the memconductance increase/decrease curves of the AOC memristor and Ti/ZnO/Pt were much smoother than those of the Cu/ZnO/Pt and electroformed Au/ZnO/Pt. This could be attributed to the purely electronic memristive switching mechanism of the AOC memristor and Ti/ZnO/Pt. Moreover, we found that the cycle-to-cycle and device-to-device variations for the AOC memristor were much smaller than those for the other three devices (Fig. 3 and S9–S12). Given that the functional layers of these four memristive devices were deposited at the same parameters, it could be deduced that the AOC memristor has a better memconductance tuning performance than the electrically controlled memristors based on switching mechanisms of both nanofilament rupture/rejuvenation and carrier trapping/detrapping. As for the Ti/ZnO/Pt, the global increase in memconductance during successive cycling likely resulted from the generated Joule heat given the rather high programming voltage and current (Fig. S10a, c), whereas large variations in the initial memconductance among the devices might be due to various extents to which Ti reacted with ZnO (Fig. S10d) [30].

We propose that the superior memconductance tuning performance of the AOC memristor could be attributed to its purely electronic memconductance tuning mechanism as well as an extremely small amount of heat generated during the programming process given a very low power density (≈ 30 *μ*W/cm^2^) of the programming light. To verify that such weak irradiation did not induce microstructure change in the AOC memristor, we exposed the device to 350 nm light with a power density of 36 *μ*W/cm^2^ for a period of time as long as one hour, followed by measuring its memconductance increase/decrease cycles (Fig. S13). By comparing Fig. 3b and S13, we were unable to observe any obvious performance deterioration after this long-term irradiation. Generally, optoelectronic characteristics of a semiconductor device are extremely sensitive to its microstructure change. Hence, the microstructure change could be excluded in the programming process of this AOC memristor given that the shortest wavelength used in our experiments was 350 nm.

It is worth mentioning that the basic optoelectronic performance of the Au/ZnO/Pt device was irrespective of the polarity of read voltage. As shown in Fig. S14a–c, when a read voltage of –10 mV was used, the device demonstrated similar optical SET and RESET behaviors as well as nonvolatility of the light-induced memconductance states to the case of using 10 mV read voltage. In Fig. S14b, we could observe a sudden increase in the absolute value of current upon 650 nm irradiation, which was likely due to the generation of a photovoltaic voltage (Fig. S14d).

### 3.4 Nonvolatile neuromorphic computing

**Fig. 4:**
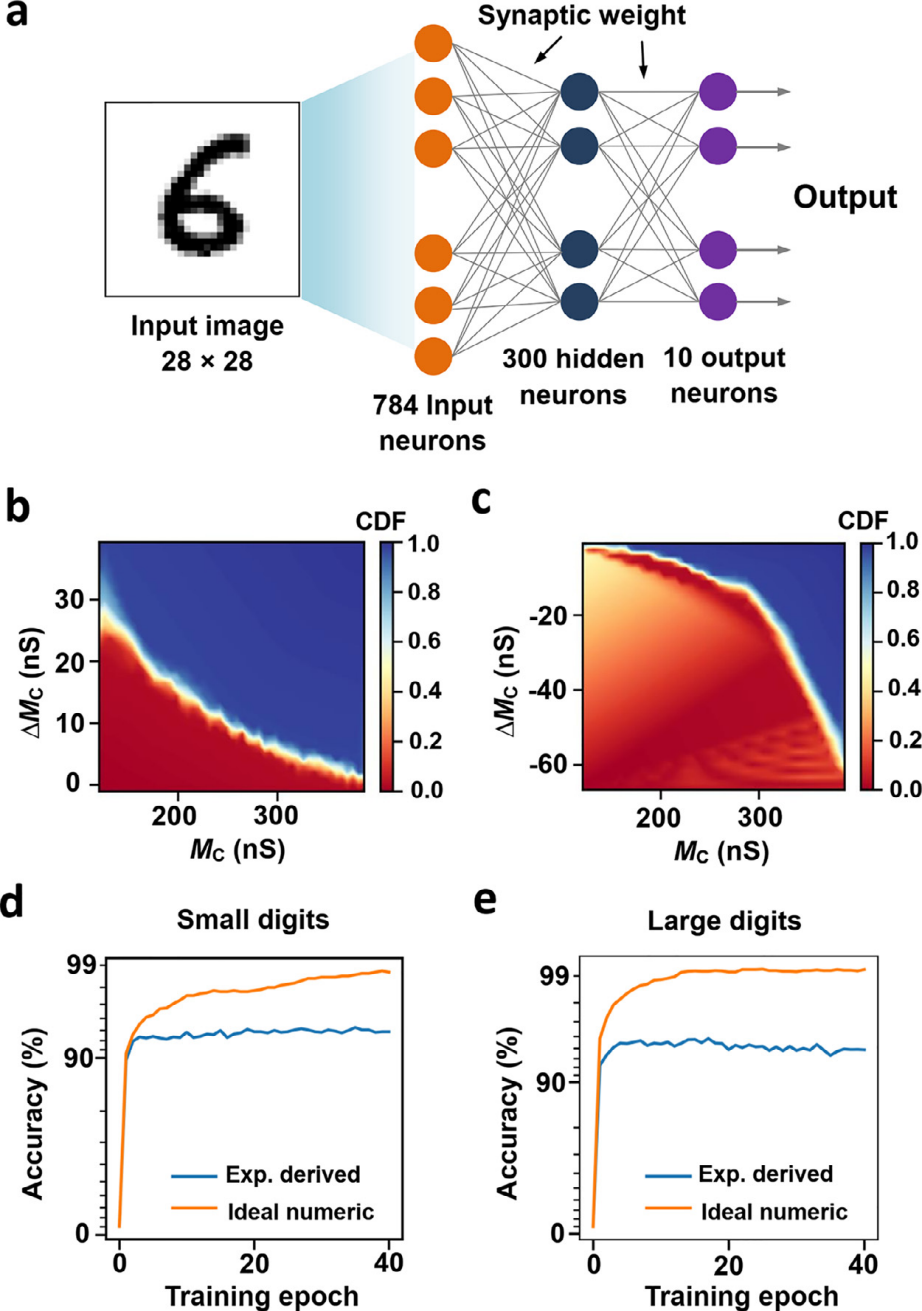
**Image recognition simulations.**(a) Schematic illustration of a three-layer ANN for recognition of 28 × 28 pixel images of handwritten digits. (b,c) ∆*M*_C_ versus *M*_C_ statistics of the AOC memristor for the SET (b) and RESET (c) processes in Fig. 3b. The heat maps encompass data from 2000 measurements. The color represents the CDF of ∆*M*_C_ at each memconductance state. (d,e) Training results based on 8 × 8 pixel images of handwritten digits (d) and 28 × 28 pixel images of handwritten digits (e).

Our AOC memristor could be used to perform nonvolatile neuromorphic computing, for example, matrix‒vector multiplication. As schematically illustrated in Fig. 4a, a three-layer artificial neural network (ANN) was constructed with the CrossSim [42] simulator using the measured memconductance values in Fig. 3b as synaptic weights. The ANN could be utilized to recognize handwritten digits. The following two datasets were employed to train the ANN: small images (8 × 8 pixels) of handwritten digits from the “Optical Recognition of Handwritten Digits” dataset [43] and large images (28 × 28 pixels) of handwritten digits from the “Modified National Institute of Standards and Technology” dataset [44]. During the training process, synaptic weights were updated based on a back-propagation algorithm. Fig. 4b and c shows the cumulative distribution functions (CDFs) for optical SET and optical RESET processes in Fig. 3b. Herein, CDF is the probability that ∆*M*_C_ takes a value less than or equal to the ∆*M*_C_ plotted. During training, the CDF was randomly sampled for weight update of each synapse. The training results for two datasets are illustrated in Fig. 4d, e (blue curves). We could observe that after three epochs, the recognition accuracy for both small and large images of handwritten digits exceeded 92%. The yellow curves in Fig. 4d, e show the simulation results of the ideal floating-point-based ANN (theoretical limit for the algorithm). The recognition accuracy of the ideal ANN exceeded 98% after 40 epochs, indicating promising application prospects of our AOC memristor in image recognition.

### 3.5 Complete Boolean logic functions

**Fig. 5:**
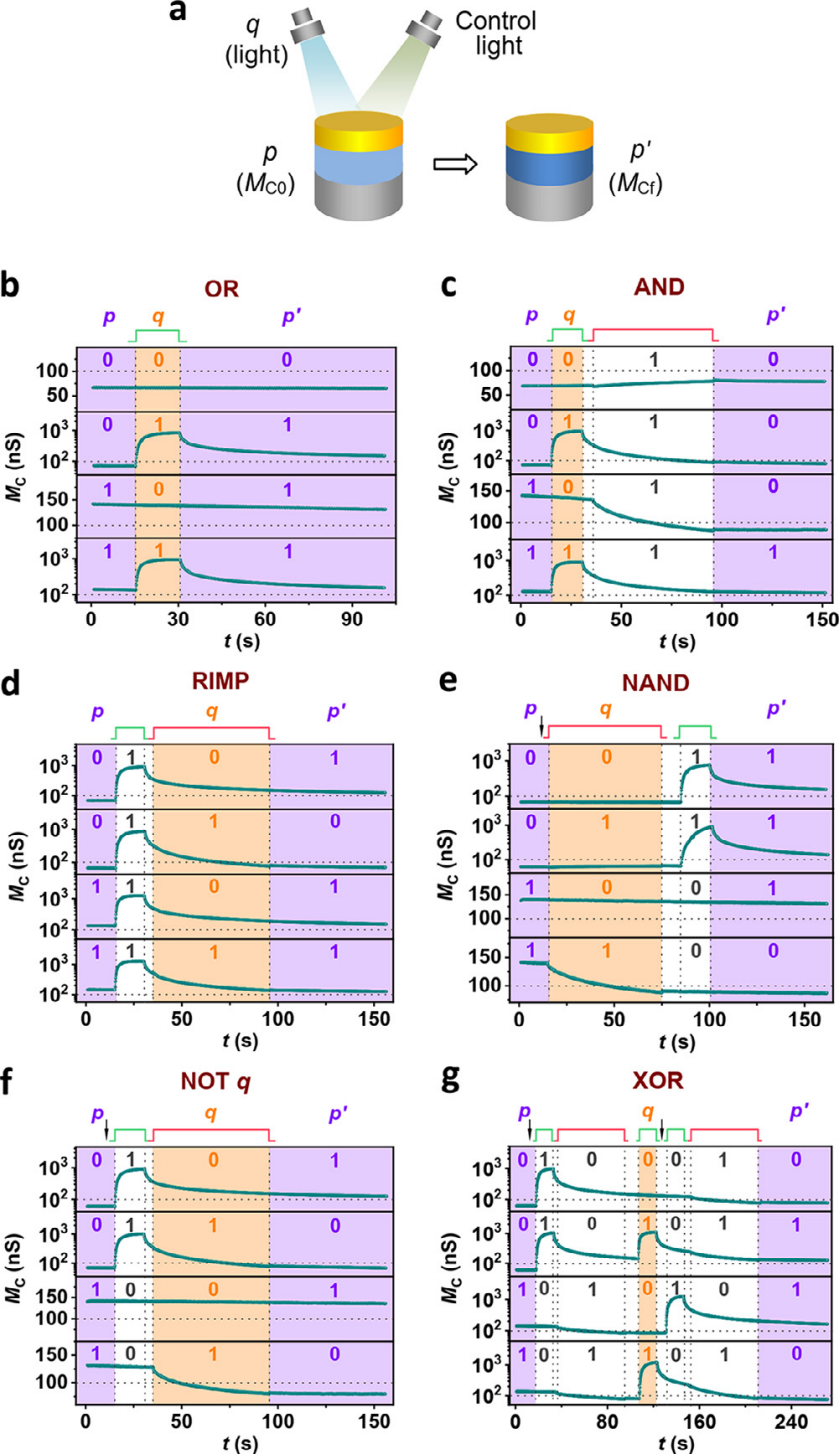
**Nonvolatile logic computing in the AOC memristor.**(a) Schematic of the realization of logic functions. *p* and *q* are the inputs and *p*ʹ is the output. *M*_C0_ and *M*_Cf_ are the initial and final memconductances, respectively. In most cases, control light is required. (b–g) OR, AND, RIMP, NAND, NOT *q*, and XOR logic functions. The green and red polylines represent 530 nm and 650 nm light, respectively. The black arrows indicate the steps of measuring *M*_C0_ and intermediate memconductance. The memconductance values were measured at 10 mV.

Boolean logic is a form of algebra in which the variable's values are the truth values, *i.e.*, true and false (generally denoted as “1” and “0”, respectively). It fits well with the binary numbering system used by modern computers, where each bit has a value of either 1 or 0. There are 16 Boolean logic functions in two-input (*e.g., p* and *q*) systems. Compared to traditional logic gates based on transistors, logic-in-memory computing architectures provide a more efficient way to store and process information [45,46]. Nonvolatile Boolean logic could be demonstrated in our AOC memristor, in which the computing results were *in situ* stored as the memconductance states. As schematically illustrated in Fig. 5a, one input *p* is related to the initial memconductance *M*_C0_ whereas the other input *q* is related to the light of 530 nm or 650 nm. The output *p*ʹ depends on the final memconductance *M*_Cf_. Except for the OR and NIMP functions, control light of 530 nm or 650 nm was also needed to demonstrate the other 14 logic functions, *i.e.*, input light and control light have to be applied to the device in certain sequences to obtain required outputs. Herein, 100 nS was set as the baseline value of memconductance; that is, *p* or *p*ʹ = 1 meant *M*_C0_ or *M*_Cf_ > 100 nS and *p* or *p*ʹ = 0 meant *M*_C0_ or *M*_Cf_ < 100 nS. *M*_C0_ was set to be about 140 nS (*p* = 1) or 70 nS (*p* = 0). *q* equals 1 (or 0) when the input light was on (or off). The same rule applied to the control light. In some cases, the control light was applied prior to the input light, referred to as the optoforming operation.

According to the operation complexity, 16 logic functions could be classified in six categories, *i.e.*, i) only needing input light (Fig. 5b and 15a), ii) needing control light (Fig. 5c and S15b, c), iii) needing an optoforming process (Fig. 5d and S15d), iv) needing measurement of *M*_C0_ (Fig. 5e and S15e–h), v) needing measurement of *M*_C0_ and optoforming processes (Fig. 5f and S15i), vi) needing measurement of *M*_C0_, optoforming processes, and measurement of intermediate memconductance (Fig. 5g and S15j).

Specifically, the OR and NIMP functions could be implemented with or without 530 nm and 650 nm light irradiation, respectively; the wavelength of input light was determined by the logic operation type. The AND, TRUE, and FALSE functions were demonstrated by sequentially applying the input light and the control light; wavelength of the control light depends on the logic operation type and the input light. For the RIMP and *q* functions, prior to the input light, the device was exposed to 530 nm and 650 nm light and set to an HMS and LMS, respectively; wavelength of the control light was selected according to the logic operation type. As for the NAND, IMP, RNIMP, *p*, and NOT *p* functions, the schemes of applying the control light strongly depend on the initial memconductance state (*p* = 1 or 0), which should be determined first, as well as the logic operation type and the input light. In the case of NOT *q* and NOR functions, the optoforming operations were necessary; the schemes of applying the control light were determined by the initial memconductance state and logic operation type. Other than the above 14 logic functions, additional steps were needed to achieve the XOR and NXOR functions. Apart from initial state-dependent optoforming processes, it was necessary to determine the intermediate memconductance states prior to applying the subsequent control light. Furthermore, the control light applied before and after the input light was composed of both 530 nm and 650 nm irradiation.

As mentioned previously, memconductance decay occurs after light irradiation. In the case of *p*ʹ = 0, such decay did not affect the device state since the memconductance gradually deviated away from the baseline value (100 nS). On the other hand, for *p*ʹ = 1, the memconductance gradually approached the baseline value as a result of the decay. To confirm the nonvolatility of the output, the decay curves were fitted using exponential functions (Fig. S16). The fitting results show that the memconductance could maintain its relatively large value above 100 nS over time, thus indicating the nonvolatile output.

## 4 Conclusion

An AOC memristor with a simple Au/ZnO/Pt structure was fabricated. The memconductance could be reversibly tuned over a continuous range via varying only the wavelength of the controlling light. The device could be operated at light power densities as low as ≈ 30 *μ*W/cm^2^ and the light-induced memconductance states were found to be nonvolatile. The observed memconductance switching behavior most likely stemmed from a reversible curvature variation of the conduction band of ZnO due to electron trapping and detrapping at oxygen vacancies. The device showed excellent operation stability. Advanced computing such as nonvolatile neuromorphic computing and complete Boolean logic functions was demonstrated using this AOC memristor, indicating its great potential as a candidate for the next generation of artificial visual systems with the capability of real-time information sensing, processing and storage.

It deserves mentioning that before AOC memristive ISCM devices could be practically used, an answer to the following question must be found, *i.e*., how can light be controllably introduced into each device to tune its memconductance in a memristor crossbar array? Usage of thin-film optical waveguides based on Si/SiO_2_, Si_3_N_4_/SiO_2_, *etc*., might be a feasible and effective method to overcome this challenge. These waveguide materials are easy to be integrated into memristive devices, making high-density integration of AOC ISCM devices possible.

## Declaration of competing interest

The authors declare that they have no conflicts of interest in this work.

## Appendix Supplementary materials

Image, application 1
