# Supplementary material for: Optically driven intelligent computing with ZnO memristor
Source: Fundam Res. 2022 Jul 25;4(1):158–66. doi: 10.1016/j.fmre.2022.06.019 (PMC11197590; doi:10.1016/j.fmre.2022.06.019)
Supplement: Supplementary file 1 [file mmc1.pdf]

Supplementary materials

Optically driven intelligent computing with ZnO memristor

Jing Yang[a,1], Lingxiang Hu[a,b,1], Liufeng Shen[a], Jingrui Wang[a], Peihong Cheng[a], Huanming Lu[a,*], Fei Zhuge[a,b,c,d,*], and Zhizhen Ye[d,e]

[a] *Ningbo Institute of Materials Technology and Engineering*
Chinese Academy of Sciences
Ningbo 315201, China

[b] *Center of Materials Science and Optoelectronics Engineering*
University of Chinese Academy of Sciences
Beijing 100029, China

[c] *Center for Excellence in Brain Science and Intelligence Technology*
Chinese Academy of Sciences
Shanghai 200072, China

[d] *Institute of Wenzhou*
Zhejiang University
Wenzhou 325006, China

[e] *State Key Laboratory of Silicon Materials*
School of Materials Science and Engineering
Zhejiang University
Hangzhou 310027, China

*Corresponding authors: hmlu@nimte.ac.cn (L.H.); zhugefei@nimte.ac.cn (F.Z)
[1] These authors contributed equally.

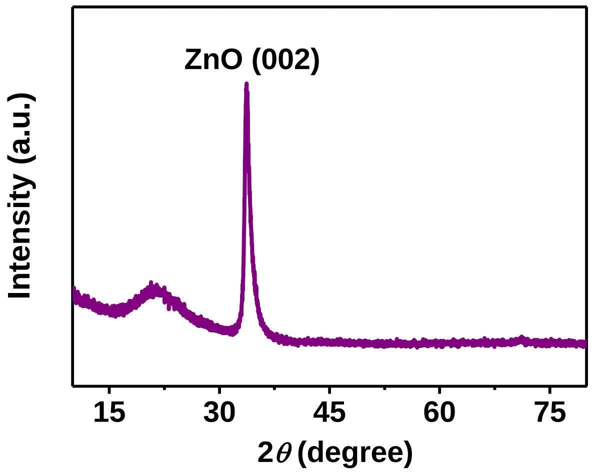

Fig. S1. XRD pattern of the ZnO thin film deposited on quartz.

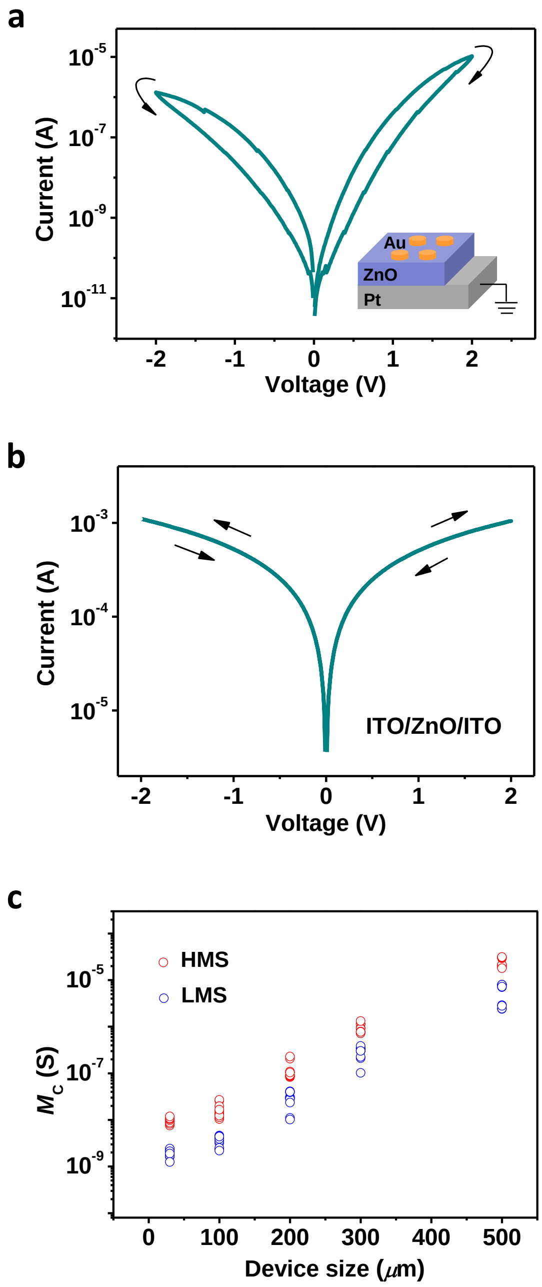

Fig. S2. Nonpolar memristive switching behavior. (a) Current–voltage curves. (b) Current–voltage curves of ITO/ZnO/ITO. (c) Dependence of the memconductance of the Au/ZnO/Pt memristor on device size. For each size, 10 memconductance values of the HMSs and LMSs obtained from 10 randomly selected devices are plotted. The memconductance values were obtained at 10 mV.

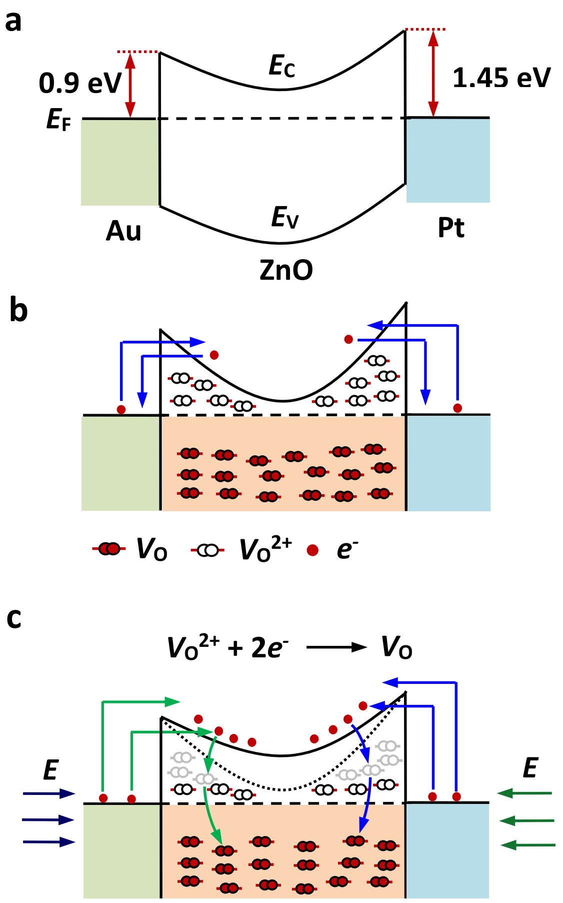

Fig. S3. Memristive switching mechanism. (a) Schematic illustration of the Au/ZnO and ZnO/Pt Schottky junctions. If ignoring interface states, the Schottky barrier heights could be estimated from the differences between the electron affinity of ZnO (4.2 eV) and the work functions of Au (5.1 eV) and Pt (5.65 eV). If taking no account of the ZnO film thickness, the Schottky barrier widths of Au/ZnO and Pt/ZnO could be estimated to be 1.96 and 2.69 μm, respectively, according to $(2\varepsilon_0\varepsilon_r V_D/nq)^{1/2}$, where ε_0 is the vacuum dielectric constant, ε_r the relative dielectric constant of ZnO (7.9), V_D the contact potential difference between metal and ZnO, n the electron concentration of ZnO, and q the elementary charge. V_D could be calculated by $(W_m - W_s)/q$, where W_m and W_s are the work functions of metal and ZnO (4.47 eV) [S1], respectively. E_F, E_C, and E_V denote the Fermi energy, the conduction band minimum, and the valence band maximum, respectively. (b) Equilibrium energy band diagram corresponding to the Au/ZnO/Pt device in the pristine state. (c) Equilibrium energy band diagram after applying a bias voltage or an electric field E (navy or dark green arrows). The Schottky emission and tunneling processes of electrons in the Au or Pt electrode as well as the subsequent V_O^{2+} neutralization reaction are also schematically illustrated (green or blue arrows). The black dotted line indicates the positions of E_C before applying the bias voltage. It deserves mention that both the electron motion and V_O^{2+} neutralization reaction actually occur during nonequilibrium conditions.

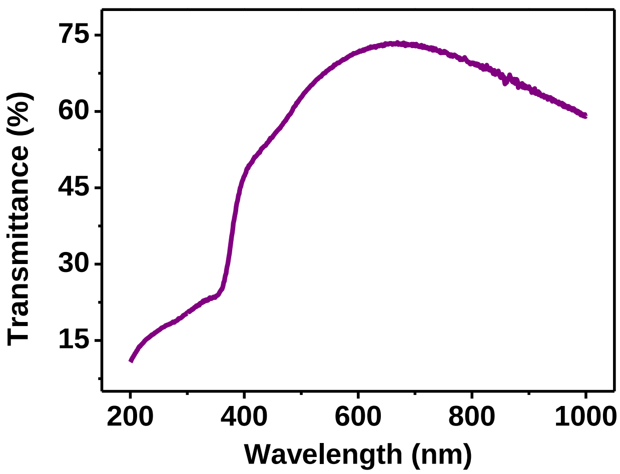

Fig. S4. Optical transmittance spectrum of Au/ZnO. Thickness of Au is 10 nm.

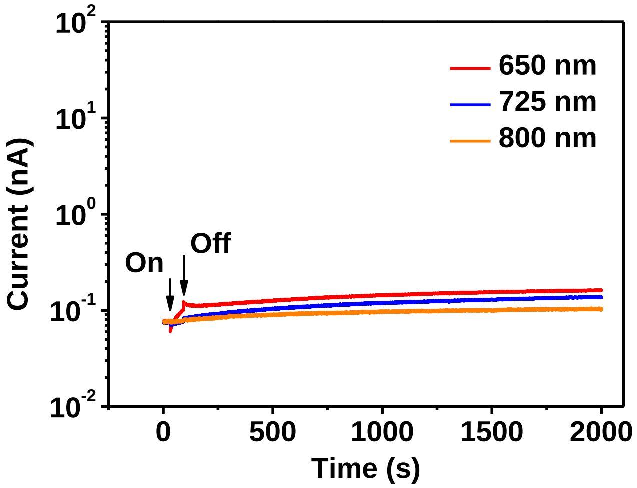

Fig. S5. Photocurrent responses of the AOC memristor to irradiation with light of 650, 725, and 800 nm. The irradiation duration was 60 s. The current values were measured at 10 mV.

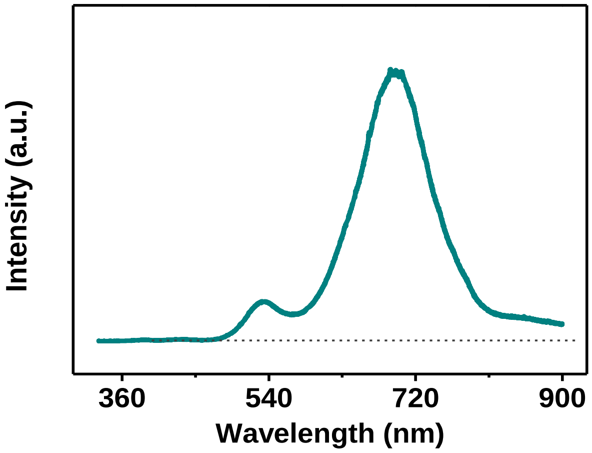

Fig. S6. Photoluminescence spectra of the ZnO thin film.

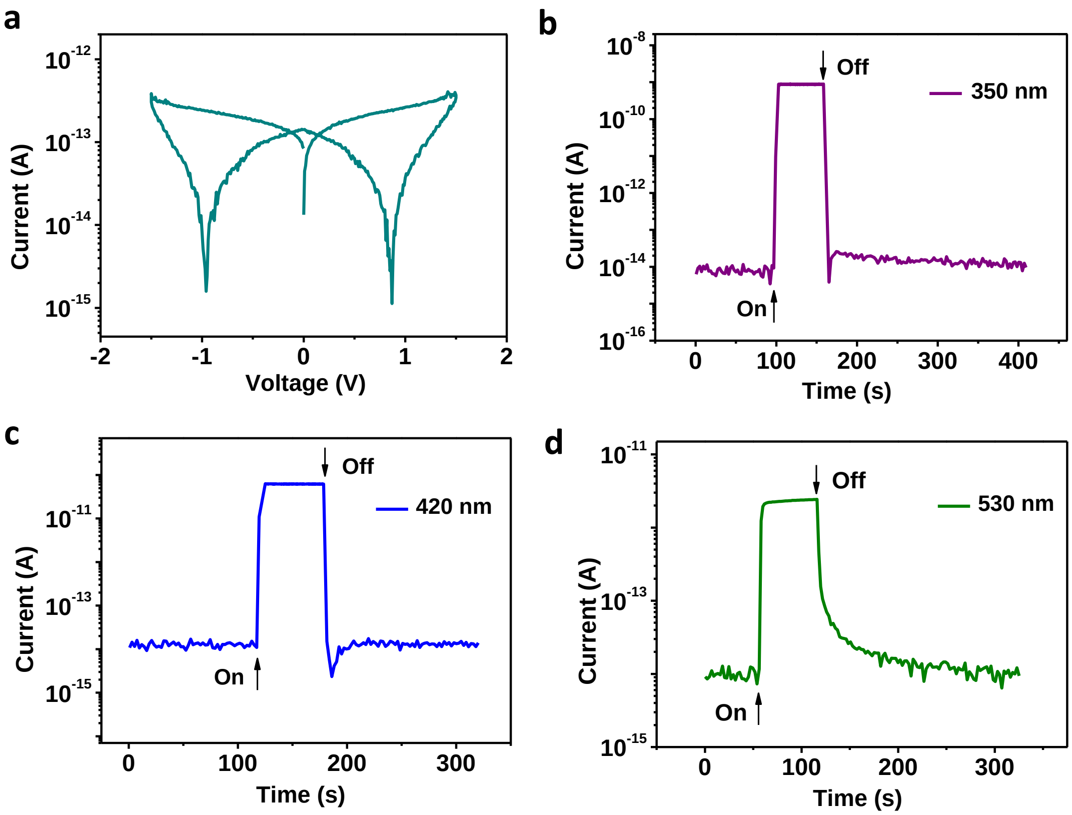

Fig. S7. Electrical and optoelectronic behaviors of Au/ZnO/Pt with ZnO deposited at an Ar/O_2 mixture atmosphere (14% O_2). (a) Current–voltage curves. (b-d) Photocurrent responses of Au/ZnO/Pt to irradiation with light of 350 (b), 420 (c), and 530 nm (d). The irradiation duration was 60 s. The current values were measured at 10 mV.

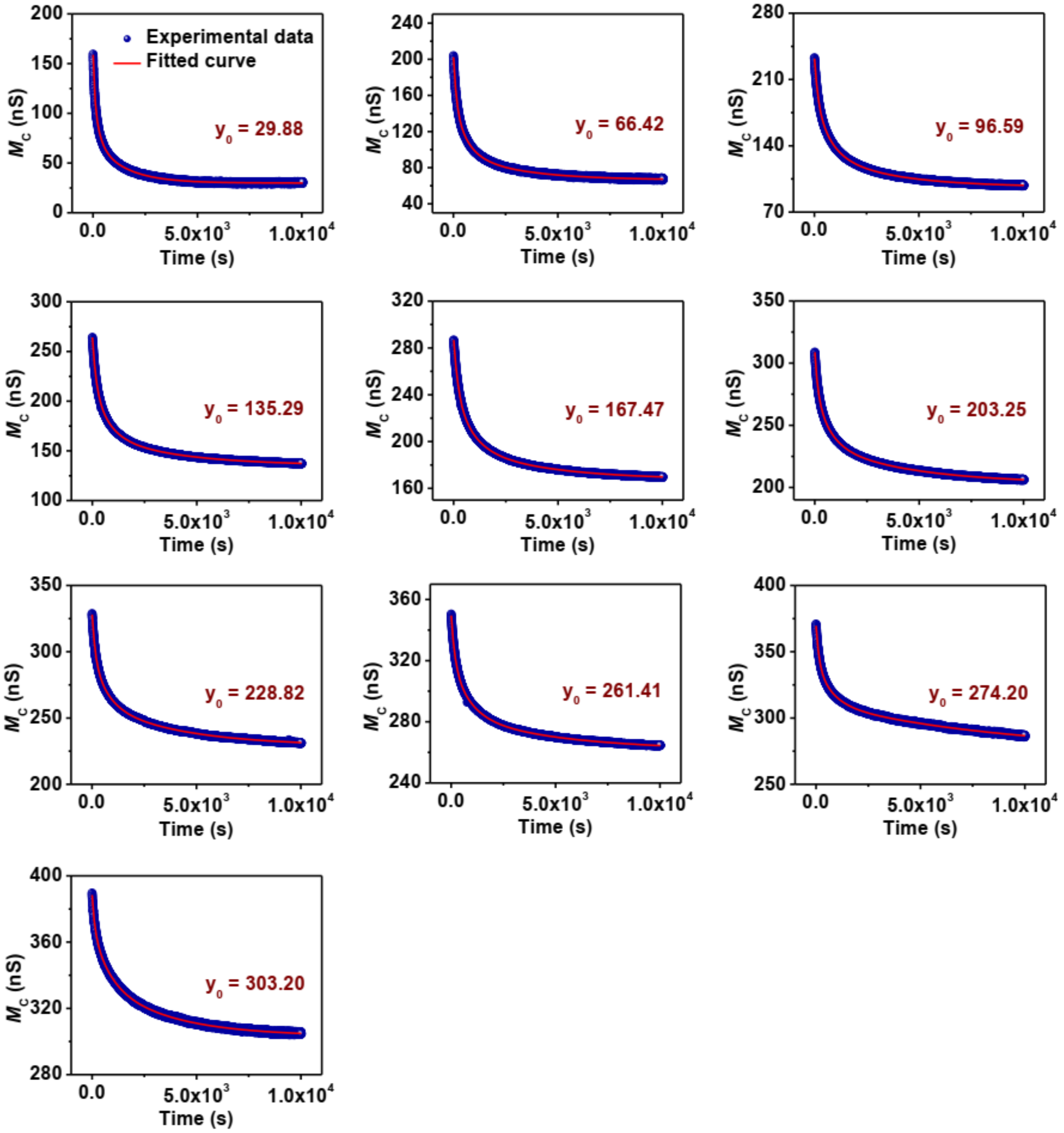

Fig. S8. Nonvolatility of the 10 memconductance states in Fig. 3a. The data was fitted by the following exponential function: $M_C = y_0 + A\exp(-t/\tau_1) + B\exp(-t/\tau_2) + C\exp(-t/\tau_3)$, where y_0, A, B, C, τ_1, τ_2, and τ_3 are positive constants. The fitting results demonstrate that the memconductance could be maintained above a certain value (y_0) over time, thus confirming the nonvolatile memconductance states.

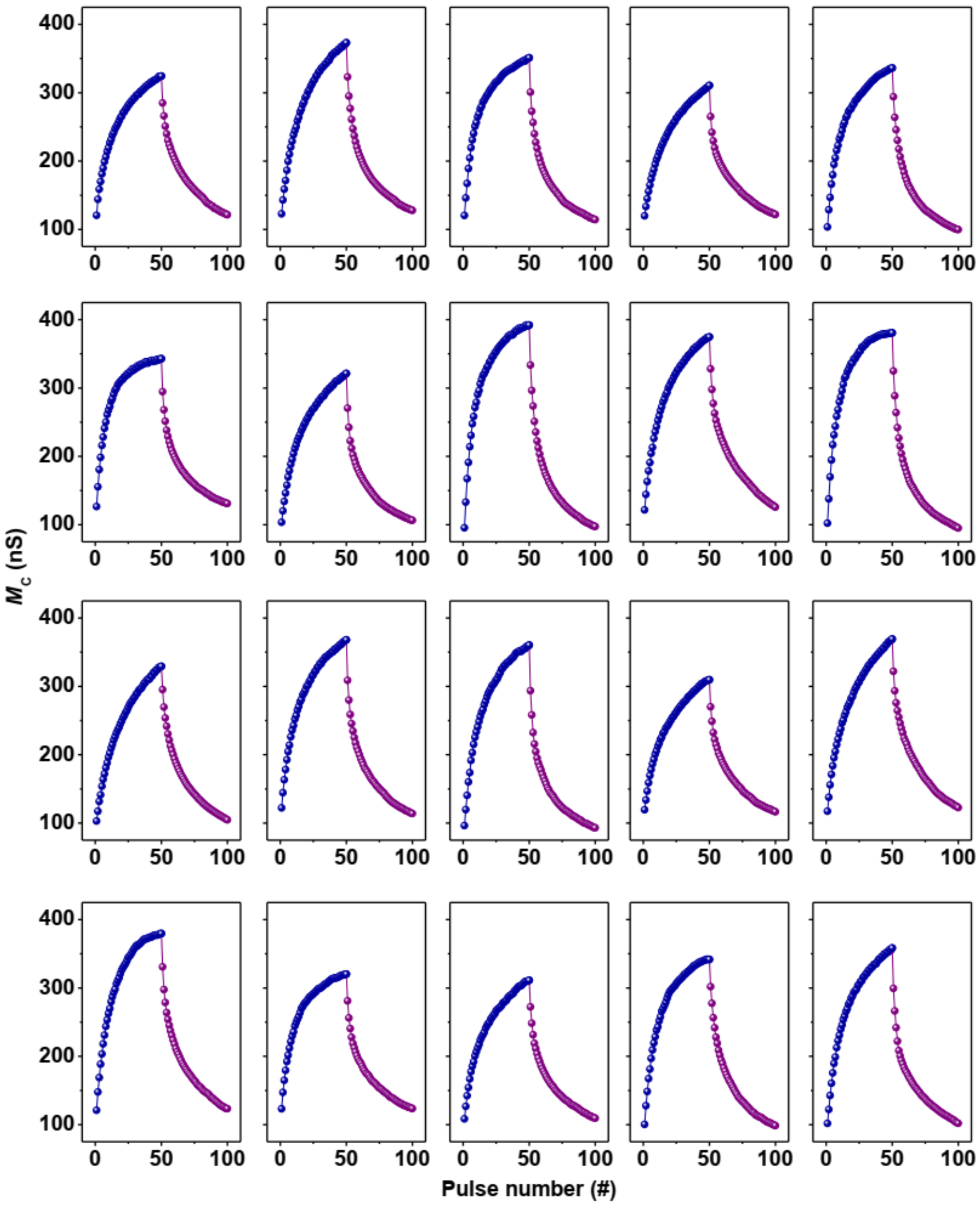

Fig. S9. Memconductance increase/decrease cycles from 20 randomly selected AOC memristive devices. The reversible modulation of memconductance was carried out by using 50 green light pulses (*D* = 100 ms and *I* = 1 s) and 50 red light pulses (*D* = 200 ms and *I* = 1 s). The memconductance values were measured 1 s after each pulse at 10 mV.

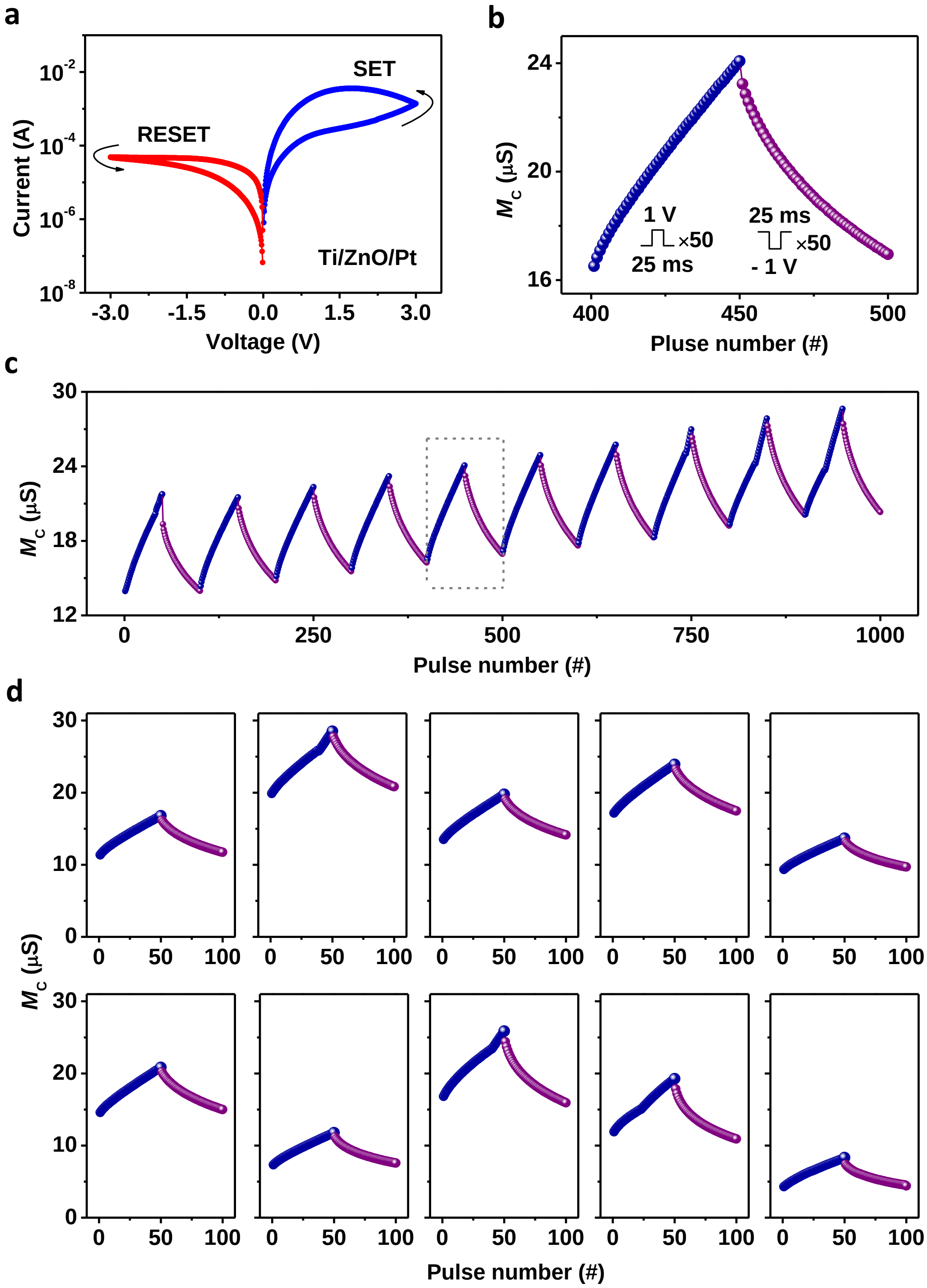

Fig. S10. Ti/ZnO/Pt memristor based on a memristive switching mechanism of electrically controlled electron trapping and detrapping. (a) Current–voltage curves. (b) Reversible modulation of memconductance by using 50 positive voltage pulses (I = 100 ms) and 50 negative voltage pulses (I = 100 ms). (c) 10 successive memconductance increase/decrease cycles. An enlarged view of the fifth cycle (gray rectangle) is shown in (a). (d) Memconductance increase/decrease cycles from 10 randomly selected devices. The memconductance values were measured 100 ms after each pulse at 10 mV.

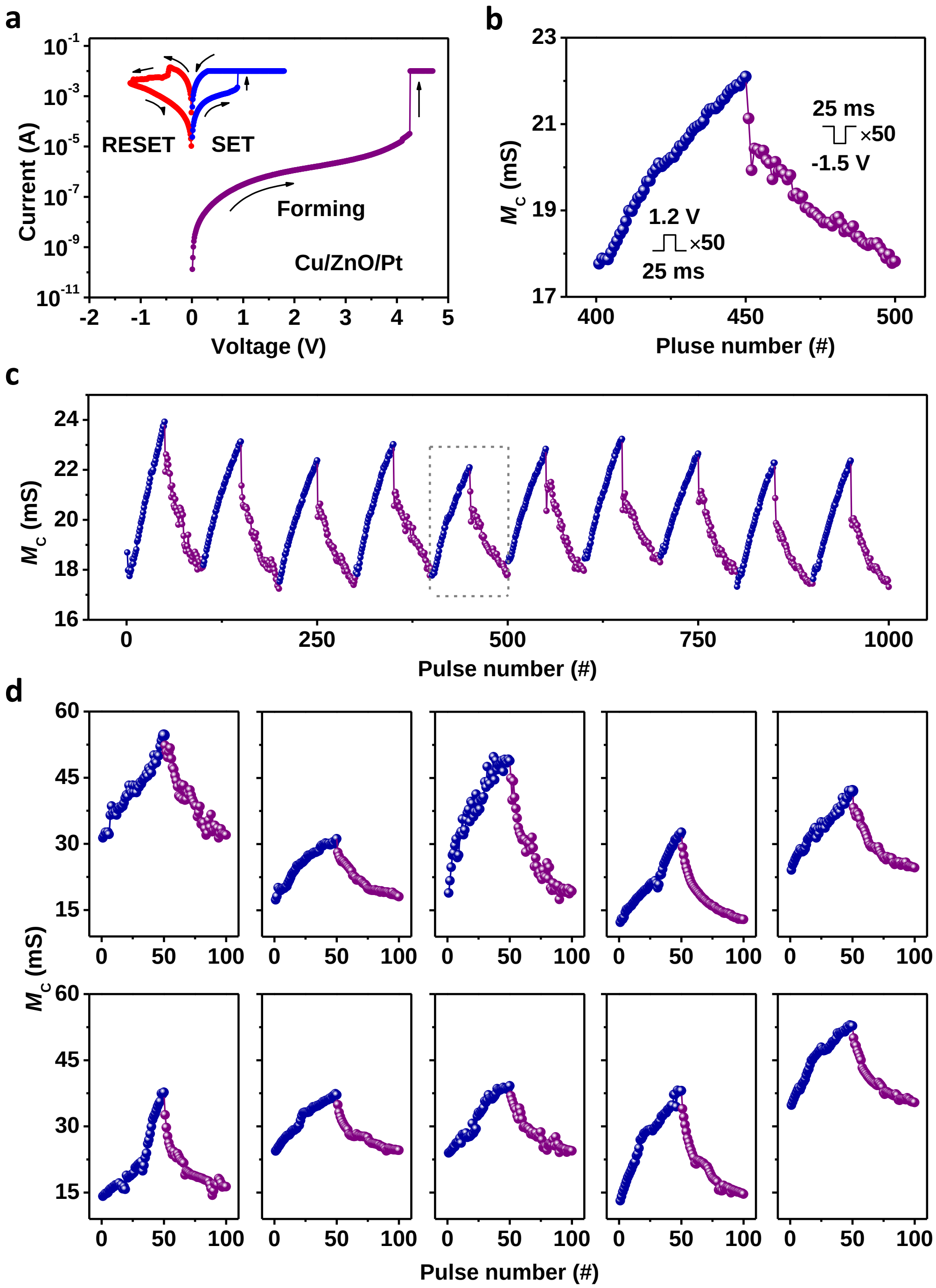

Fig. S11. Cu/ZnO/Pt memristor based on a memristive switching mechanism of electrically controlled Cu nanofilament rupture and rejuvenation. (a) Current–voltage curves. A Forming process was needed to initialize the device. (b) Reversible modulation of memconductance by using 50 positive voltage pulses (I = 100 ms) and 50 negative voltage pulses (I = 100 ms). (c) 10 successive memconductance increase/decrease cycles. An enlarged view of the fifth cycle (gray rectangle) is shown in (a). (d) Memconductance increase/decrease cycles from 10 randomly selected devices. The memconductance values were measured 100 ms after each pulse at 10 mV.

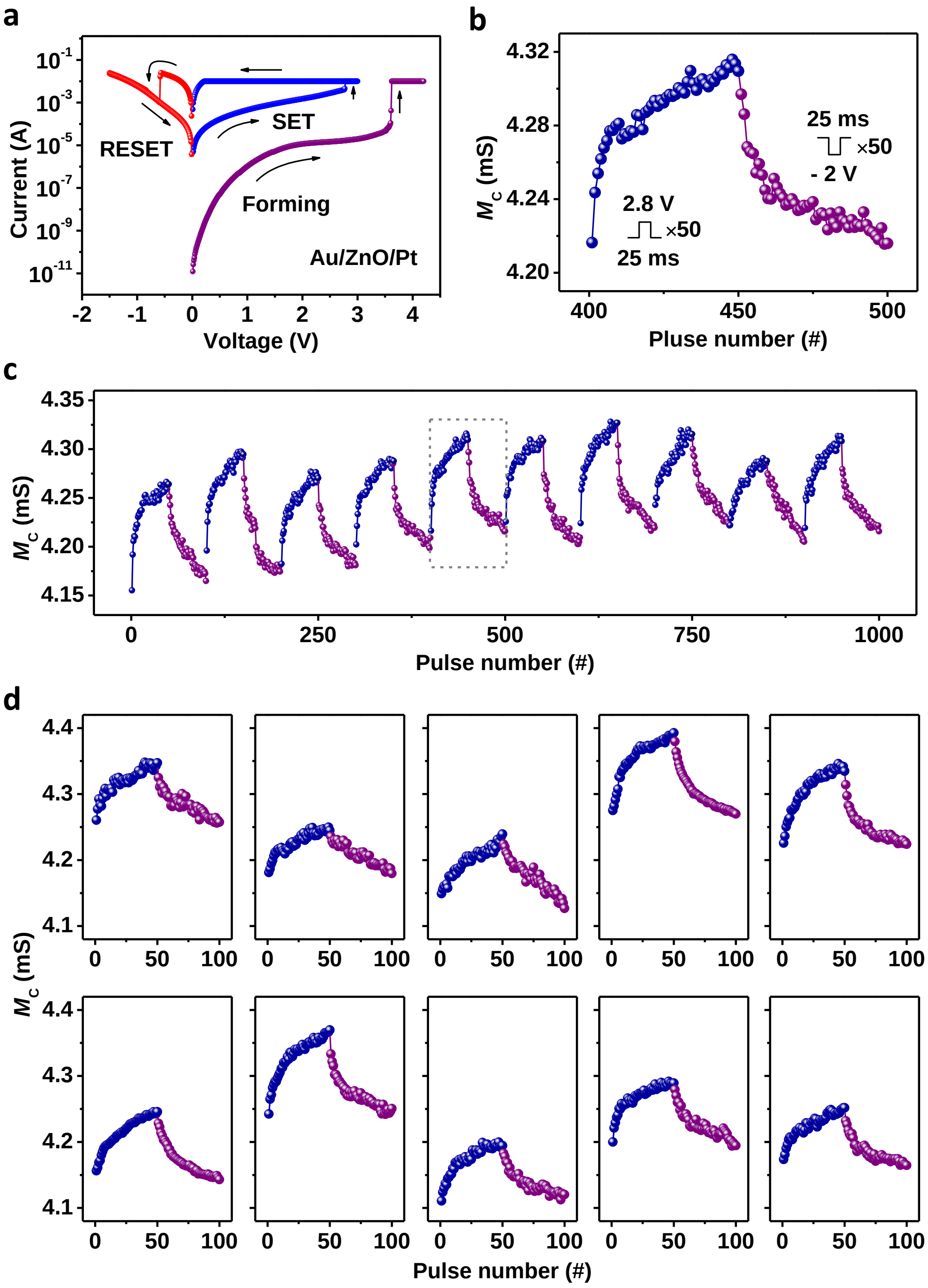

Fig. S12. Au/ZnO/Pt memristor based on a memristive switching mechanism of electrically controlled V_O nanofilament rupture and rejuvenation. (a) Current–voltage curves. A Forming process was needed to initialize the device. (b) Reversible modulation of memconductance by using 50 positive voltage pulses (I = 100 ms) and 50 negative voltage pulses (I = 100 ms). (c) 10 successive memconductance increase/decrease cycles. An enlarged view of the fifth cycle (gray rectangle) is shown in (a). (d) Memconductance increase/decrease cycles from 10 randomly selected devices. The memconductance values were measured 100 ms after each pulse at 10 mV.

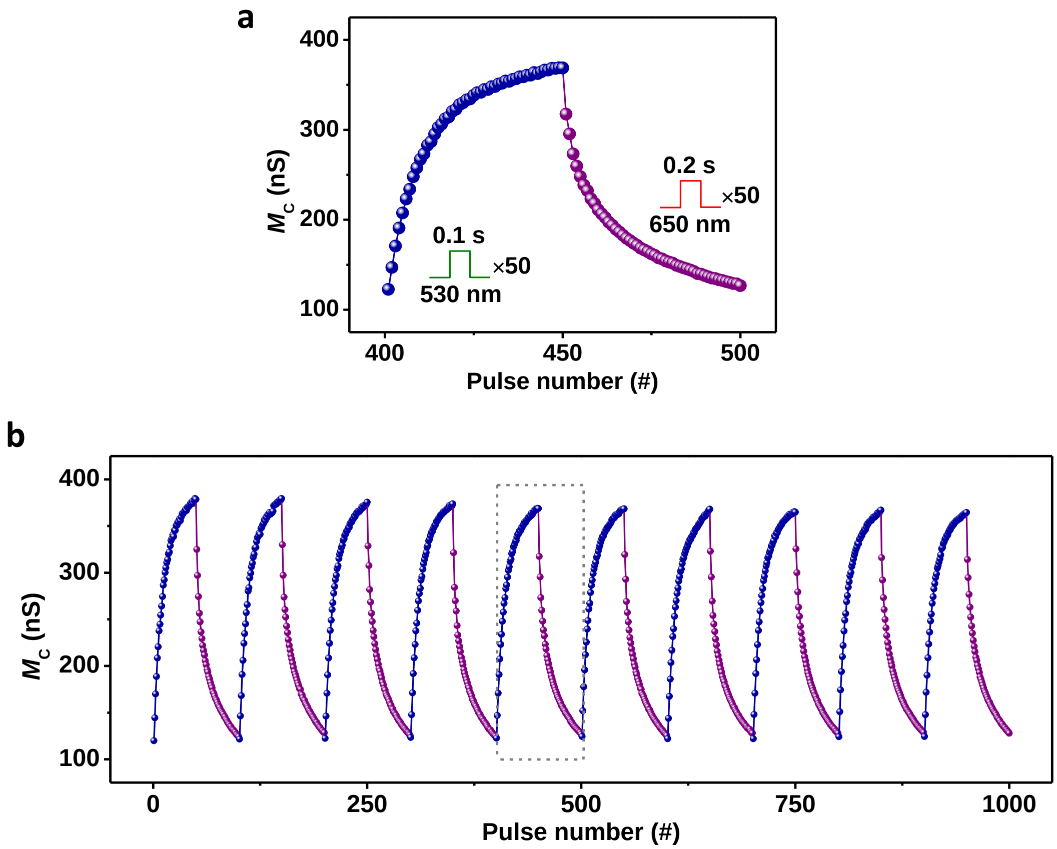

Fig. S13. Memconductance tuning performance of the AOC memristor exposed to 350 nm light with a power density of 36 μW/cm^2 for one hour. (a) Reversible modulation of memconductance by using 50 green light pulses (D = 100 ms and I = 1 s) and 50 red light pulses (D = 200 ms and I = 1 s). (b) 10 successive memconductance increase/decrease cycles. An enlarged view of the fifth cycle (gray rectangle) is shown in (a). The memconductance values were measured 1 s after each pulse at 10 mV.

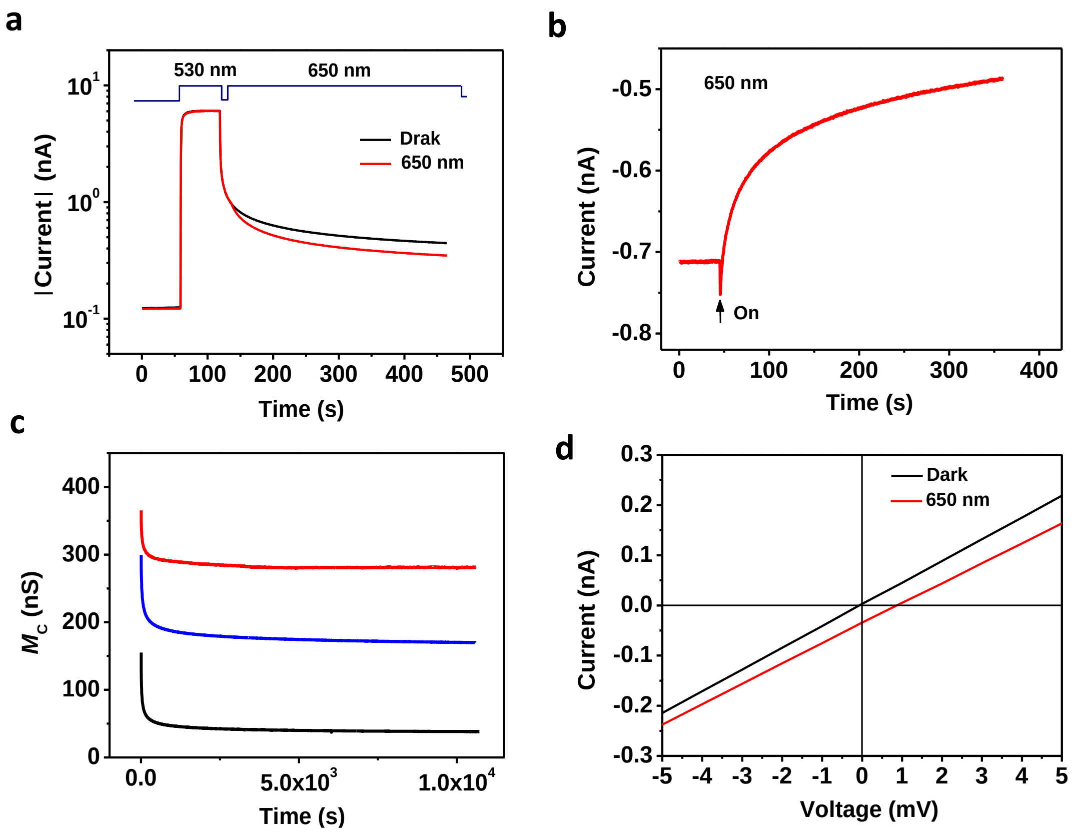

Fig. S14. Optoelectronic behavior of Au/ZnO/Pt. (a) Photocurrent response to light of 530 and 650 nm wavelengths. The device was first irradiated with 530 nm light (*D* = 60 s). (b) Optical RESET behavior upon exposure to 650 nm light (*D* = 300 s). The device was first set to a HMS by irradiating it with 530 nm light (*D* = 60 s). (c) Retention performance of three memconductance states induced by light. The device was first set to a low memconductance state followed by three SET operations with different numbers of 530 nm light pulses. After each SET operation and following retention test, the device was set to the initial low memconductance state again. (d) Current–voltage curves in dark and under 650 nm light irradiation. In (a–c), the memconductance values were measured at –10 mV.

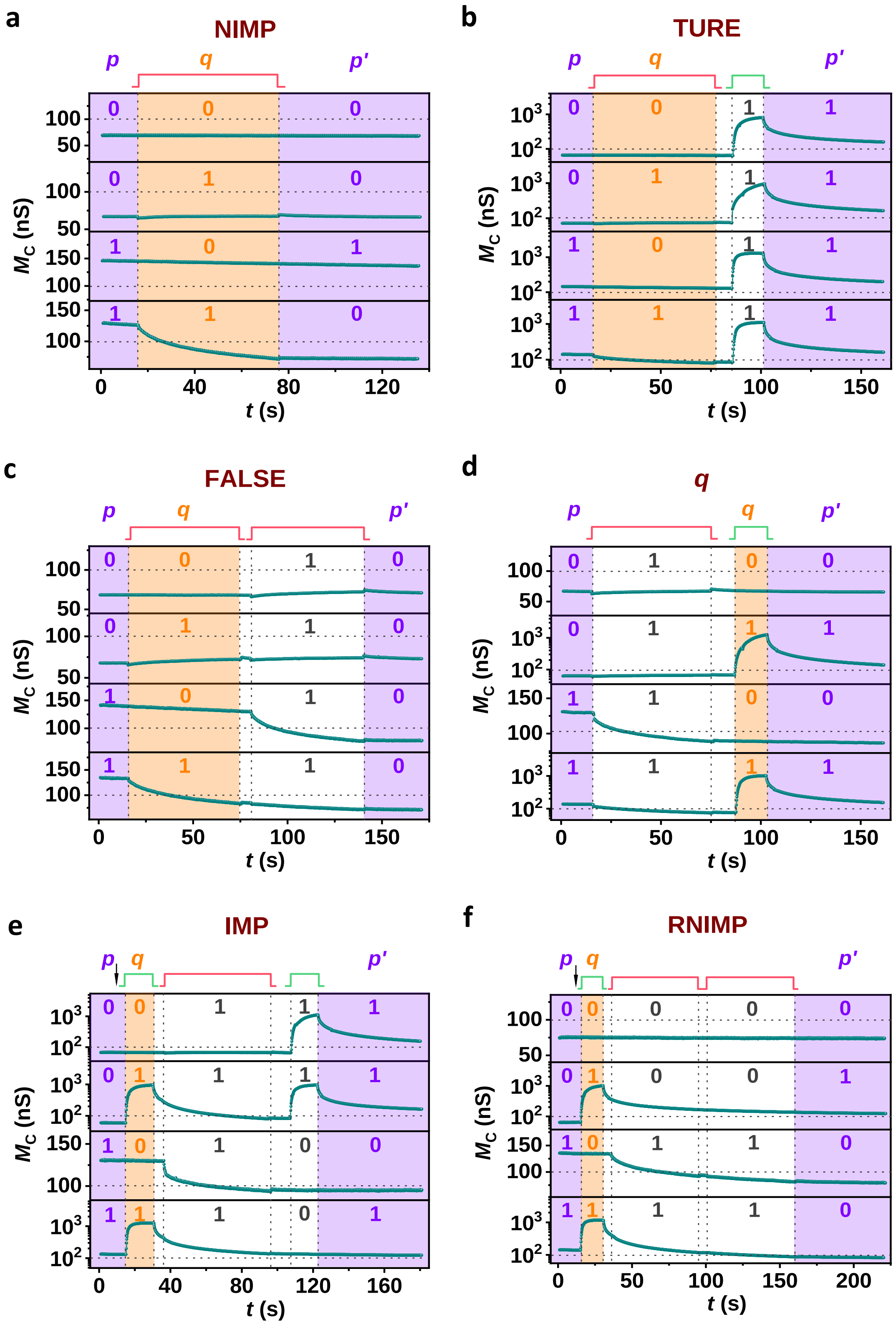
a
NIMP
b
TURE
c
FALSE
d
q
e
IMP
f
RNIMP
M_C (nS)
t (s)

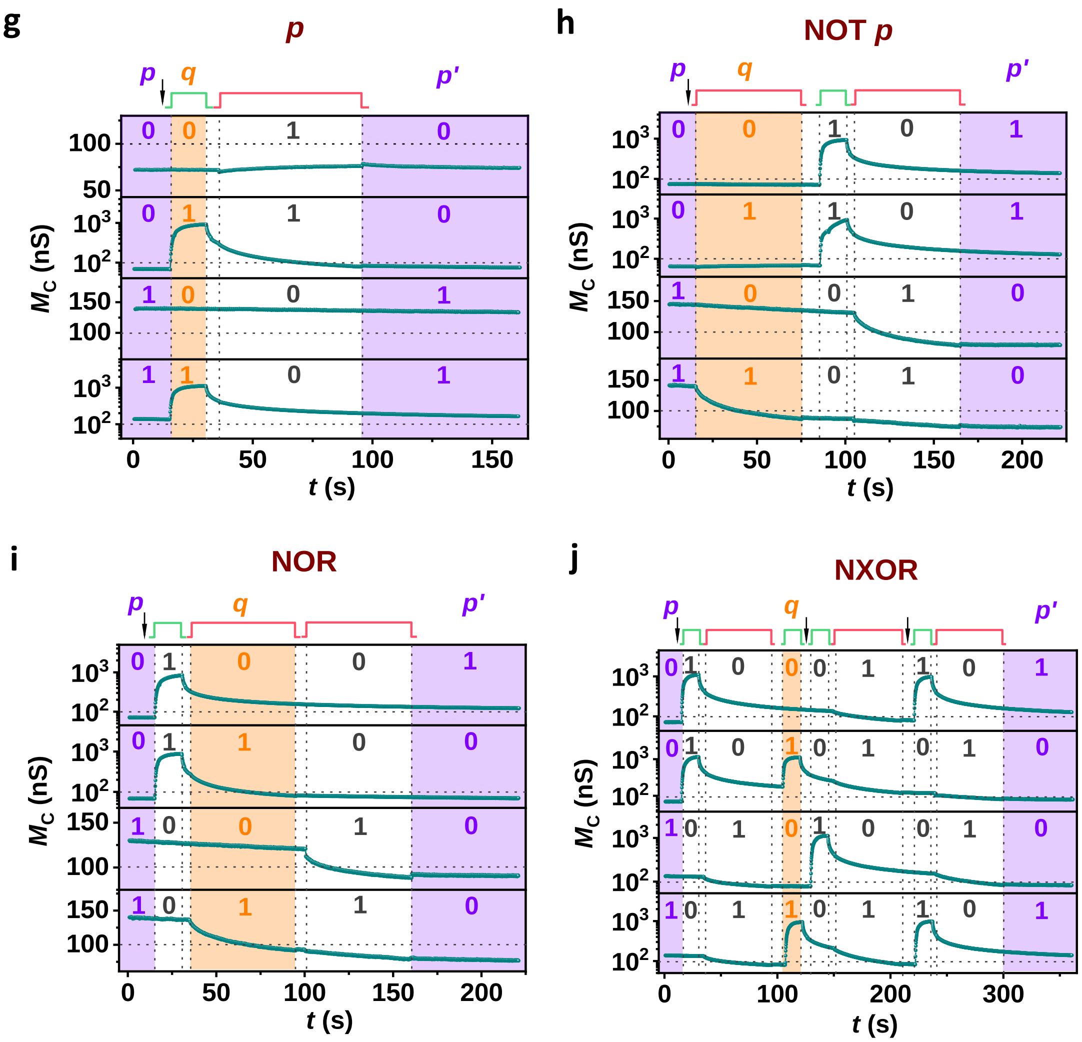

Fig. S15. Nonvolatile logic computing in the AOC memristor. (a–j) NIMP, TRUE, FALSE, *q*, IMP, RNIMP, *p*, NOT *p*, NOR, and NXOR logic functions. The green and red polylines represent 530 and 650 nm lights, respectively. The black arrows indicate the steps of measuring M_{C0} and intermediate memconductance. The memconductance values were measured at 10 mV.

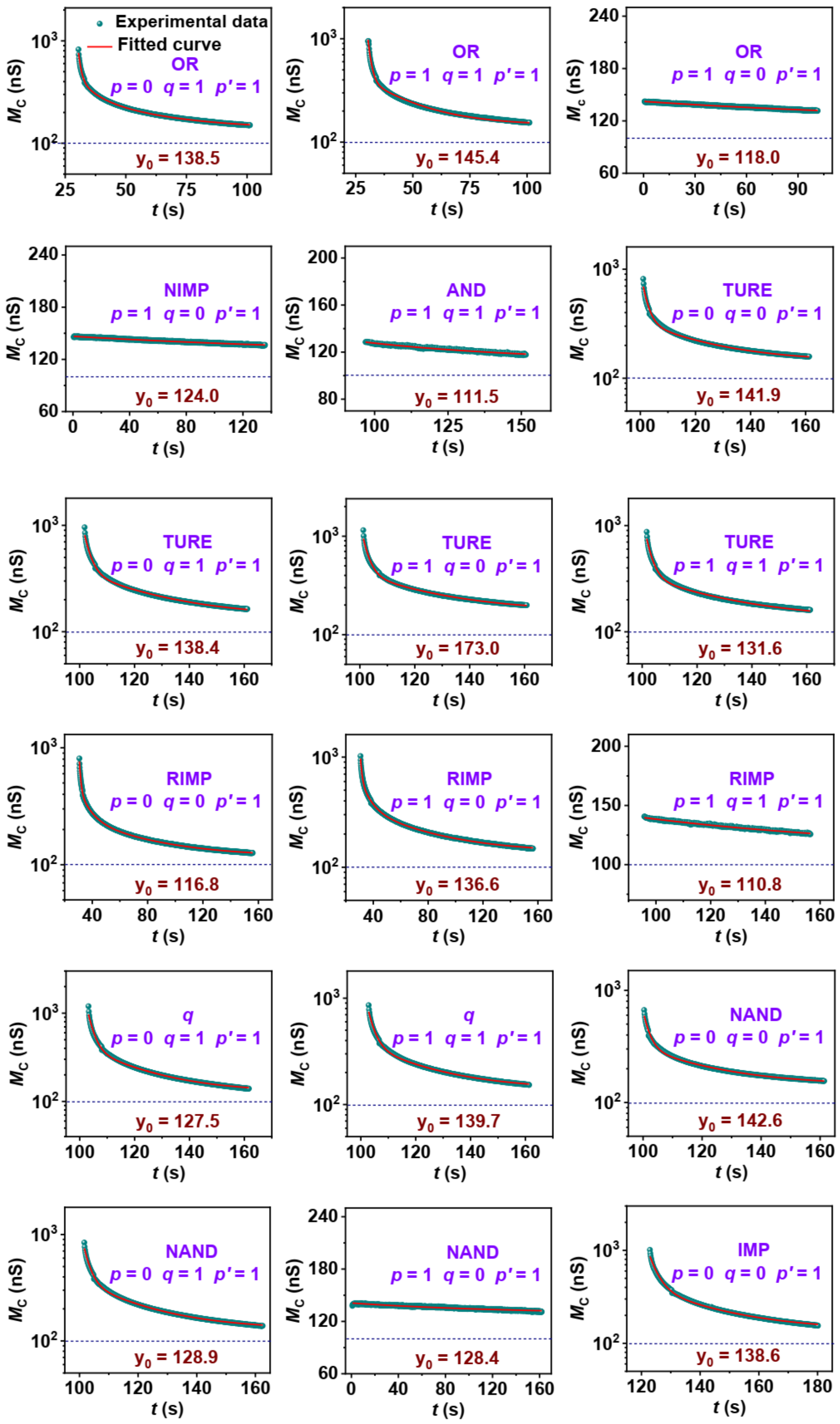

Experimental data
Fitted curve
OR
p = 0 q = 1 p′ = 1
y0 = 138.5
OR
p = 1 q = 1 p′ = 1
y0 = 145.4
OR
p = 1 q = 0 p′ = 1
y0 = 118.0
NIMP
p = 1 q = 0 p′ = 1
y0 = 124.0
AND
p = 1 q = 1 p′ = 1
y0 = 111.5
TURE
p = 0 q = 0 p′ = 1
y0 = 141.9
TURE
p = 0 q = 1 p′ = 1
y0 = 138.4
TURE
p = 1 q = 0 p′ = 1
y0 = 173.0
TURE
p = 1 q = 1 p′ = 1
y0 = 131.6
RIMP
p = 0 q = 0 p′ = 1
y0 = 116.8
RIMP
p = 1 q = 0 p′ = 1
y0 = 136.6
RIMP
p = 1 q = 1 p′ = 1
y0 = 110.8
q
p = 0 q = 1 p′ = 1
y0 = 127.5
q
p = 1 q = 1 p′ = 1
y0 = 139.7
NAND
p = 0 q = 0 p′ = 1
y0 = 142.6
NAND
p = 0 q = 1 p′ = 1
y0 = 128.9
NAND
p = 1 q = 0 p′ = 1
y0 = 128.4
IMP
p = 0 q = 0 p′ = 1
y0 = 138.6
MC (nS)
t (s)

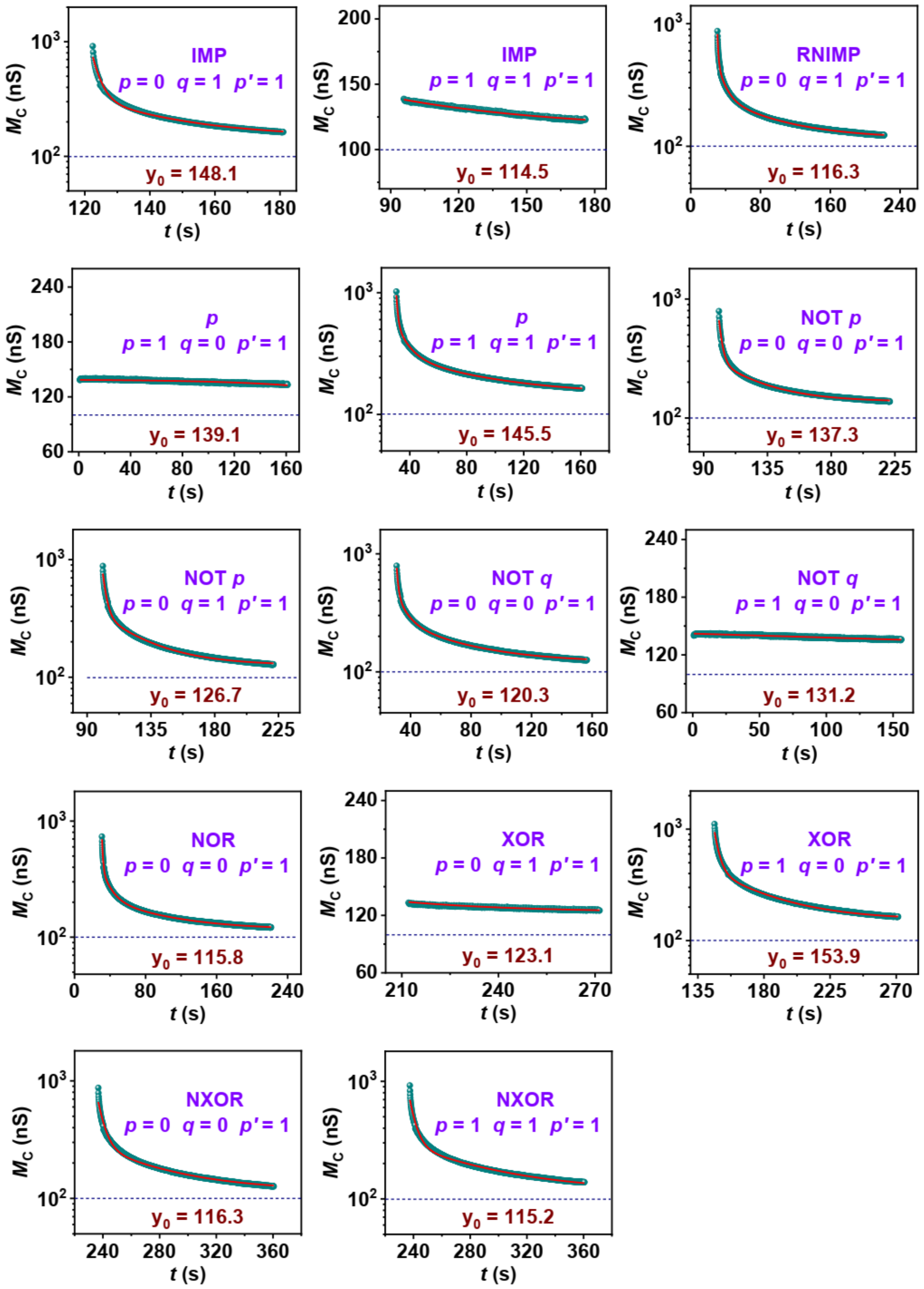

Fig. S16. Nonvolatility of the output ($p' = 1$) of the logic operations in Fig. 5 and Fig. S13. The data was fitted by the following exponential function: $M_C = y_0 + A\exp(-t/\tau_1) + B\exp(-t/\tau_2) + C\exp(-t/\tau_3)$, where y_0, A, B, C, τ_1, τ_2 and τ_3 are positive constants. The fitting results demonstrate that a relatively large value of the memconductance (above 100 nS) could be maintained over time, thus confirming the nonvolatile output.

References

[S1] K. B. Sundaram, Ashamin Khan, Work function determination of zinc oxide films, J. Vac. Sci. Technol. A 15 (1997) 428–430.
